# A transposase-derived gene required for human brain development

**DOI:** 10.1126/sciadv.adv7530

**Published:** 2026-01-14

**Authors:** Luz Jubierre Zapater, Sara A. Lewis, Rodrigo Lopez Gutierrez, Makiko Yamada, Elias Rodriguez-Fos, Merce Planas-Felix, Daniel Cameron, Phillip Demarest, Anika Nabila, Helen S. Mueller, Junfei Zhao, Paul Bergin, Casie Reed, Tzippora Chwat-Edelstein, Alex Pagnozzi, Caroline Nava, Emilie Bourel-Ponchel, Patricia Cornejo, Ali Dursun, R. Köksal Özgül, Halil Tuna Akar, Henry Houlden, Huma Arshad Cheema, Muhammad Nadeem Anjum, Giovanni Zifarelli, Peter Bauer, Miriam Essid, Hanene Benrhouma, Meriem Ben Hafsa, Ichraf Kraoua, Carolina I. Galaz-Montoya, Alex Proekt, Xiaolan Zhao, Nicholas D. Socci, Matthew Hayes, Yves Bigot, Raul Rabadan, Reza Maroofian, David Torrents, Claudia L. Kleinman, Michael C. Kruer, Miklos Toth, Alex Kentsis

**Affiliations:** ^1^ Molecular Pharmacology Program, Sloan Kettering Institute, Memorial Sloan Kettering Cancer Center, New York, NY 10021, USA.; ^2^ Tow Center for Developmental Oncology, Department of Pediatrics, Memorial Sloan Kettering Cancer Center, New York, NY 10021, USA.; ^3^ Pediatric Movement Disorders Program, Barrow Neurological Institute, Phoenix Children’s Hospital, and Departments of Child Health, Neurology, Genetics, and Cellular & Molecular Medicine, University of Arizona College of Medicine, Phoenix, AZ 85004, USA.; ^4^ Department of Human Genetics, McGill University, Montreal, Quebec, Canada.; ^5^ Barcelona Supercomputing Center (BSC), Barcelona 08034, Spain.; ^6^ Department of Pharmacology, Weill Cornell Medical College, New York, NY 10021, USA.; ^7^ Program for Mathematical Genomics, Departments of Systems Biology and Biomedical Informatics, Columbia University, New York, NY 10032, USA.; ^8^ Molecular Biology Program, Sloan Kettering Institute, Memorial Sloan Kettering Cancer Center, New York, NY 10021, USA.; ^9^ Programs in Biochemistry, Cell, and Molecular Biology, Weill Cornell Graduate School of Medical Sciences, New York, NY 10065, USA.; ^10^ Australian e-Health Research Centre, CSIRO, Brisbane, Australia.; ^11^ Sorbonne Université, Paris Brain Institute-ICM, Inserm, CNRS, Assistance Publique- Hôpitaux de Paris (APHP), Département de Génétique, Hôpital de la Pitié Salpêtrière, Paris, France.; ^12^ Research Group on Multimodal Analysis of Brain Function, University of Picardie Jules Verne, Amiens, France.; ^13^ Pediatric Neurophysiology Unit, Amiens Picardie University Hospital, Amiens, France.; ^14^ Phoenix Children’s Hospital, Phoenix, AZ 85016, USA.; ^15^ Hacettepe University, Faculty of Medicine & Institute of Child Health, Department of Pediatric Metabolism, Ankara, Turkey.; ^16^ Department of Neuromuscular Diseases, UCL Queen Square Institute of Neurology, London, UK.; ^17^ Department of Pediatric Medicine, Children’s Hospital, University of Child Health Sciences, Lahore, Pakistan.; ^18^ CENTOGENE GmbH, Rostock, Germany.; ^19^ Department of Child and Adolescent Neurology, LR18SP04, National Institute Mongi Ben Hmida of Neurology, University of Tunis El Manar, Tunis, Tunisia.; ^20^ Graduate Program in Genetics, University of Arizona, Tucson, AZ 85721, USA.; ^21^ Department of Anesthesiology and Critical Care, Perelman School of Medicine, University of Pennsylvania, Philadelphia, PA 19104, USA.; ^22^ Bioinformatics Core, Memorial Sloan Kettering Cancer Center, New York, NY 10021, USA.; ^23^ Department of Physics and Computer Science, Xavier University of Louisiana, New Orleans, LA 70125, USA.; ^24^ Physiologie de la reproduction et des comportements, UMR INRAe 0085 CNRS8247, Centre INRAE Val de Loire, Nouzilly, France.; ^25^ Institució Catalana de Recerca I Estudis Avançats (ICREA), Barcelona, Spain.; ^26^ Lady Davis Institute for Medical Research, Jewish General Hospital, Montreal, Quebec, Canada.; ^27^ Departments of Pediatrics, Pharmacology, and Physiology & Biophysics, Weill Medical College of Cornell University, New York, NY 10065, USA.

## Abstract

Vertebrate brain development is associated with prominent neuronal cell death and DNA breaks, but their causes and functions are not well understood. DNA transposable elements could contribute to somatic genome rearrangements; however, their contributions to brain development are largely unknown. PiggyBac transposable element derived 5 (PGBD5) is an evolutionarily conserved vertebrate DNA transposase–derived gene with DNA remodeling activities in human cells. Here, we show that PGBD5 contributes to normal brain development in mice and humans, and its deficiency causes disorder of intellectual disability, movement disorders, and epilepsy. In mice, Pgbd5 is required for the developmental induction of postmitotic DNA breaks and recurrent somatic brain genome rearrangements. In the cerebral cortex, loss of Pgbd5 leads to aberrant neuronal gene expression, including of specific types of glutamatergic neurons, which partly explains the features of PGBD5 deficiency in humans. Thus, PGBD5 is a transposase-derived gene required for brain development in mammals.

## Teaser

PGBD5 is required for brain development in humans and mice through genetic and nongenetic mechanisms.

## INTRODUCTION

Vertebrate brain development requires neuronal diversification and self-organization into signaling networks (*1*). While cell diversification is required for the development of many tissues, the development of nervous and immune systems is also uniquely dependent on DNA break repair and developmental apoptosis (*2*–*9*). For example, several human DNA damage repair deficiency syndromes, such as ataxia telangiectasia (AT) and Seckel syndromes, exhibit both abnormal brain neuron development and immune lymphocyte deficiencies. Likewise, mice deficient for the evolutionarily conserved DNA repair factors, such as *Xrcc5/Ku80*, are also characterized by combined abnormal neuron and lymphocyte development. In developing immune lymphocytes, efficient end-joining DNA repair is required to ligate DNA breaks induced by the domesticated DNA transposase-derived RAG1/2 during somatic diversification of immunoglobulin receptor genes (*10*).

Somatic genetic neuronal diversification of cell adhesion receptors was proposed as a mechanism for the complex organization of vertebrate brains more than 50 years ago (*11*). Initially considered for clustered protocadherins, based on their structural similarity to the immunoglobulin receptor genes (*12*, *13*), somatic DNA breaks have now been detected in a diverse set of neuronal genes (*14*, *15*). Recent single-cell sequencing studies have found extensive somatic genetic mosaicism in human neurons (*14*, *16*–*18*), bolstered by numerous prior studies in mice (*14*, *19*, *20*). While somatic DNA rearrangements and developmental apoptosis are essential for the evolution and function of vertebrate adaptive immunity, the mechanisms of somatic DNA breaks and neuronal apoptosis during brain development remain obscure.

PiggyBac transposable element derived 5 (PGBD5) is the most evolutionarily conserved domesticated DNA transposase–derived gene in vertebrates (*21*). Recently, we found that PGBD5 is expressed in most childhood solid tumors, where it mediates sequence-specific oncogenic DNA rearrangements that depend on cellular end-joining DNA repair in cells (*21*–*23*). Alanine scanning mutagenesis identified an evolutionarily conserved triad of aspartate residues in PGBD5 required for its genome remodeling activity in cells, which is reminiscent of catalytic DDD triads required for piggyBac transposase enzymes, although measurements of direct PGBD5 nuclease activity remain elusive because of the limitations of current methods for PGBD5 purification in vitro (*21*–*23*). PGBD5-induced DNA rearrangements in human cells have been corroborated by multiple laboratories (*21**,* *24**,* *25*). However, further work is needed to clarify its observed variability, as these findings were not replicated in every study, suggesting that DNA rearrangements induced by PGBD5 may involve additional mechanisms or cofactors beyond its intrinsic activities (*26*).

Since most PGBD5-expressing childhood solid tumors share a common neuroectodermal developmental origin (*22*, *25*, *27*), we hypothesized that PGBD5 may be required for normal nervous system development, as originally proposed for PGBD5’s de novo evolutionary appearance in rudimentary chordate animals by Pavelitz *et al.* (*28*), at least in part by mediating somatic DNA rearrangements in developing neurons. Here, we investigated this hypothesis using a combination of human genetic and mouse neuroscience studies.

## RESULTS

**Fig. 1.**
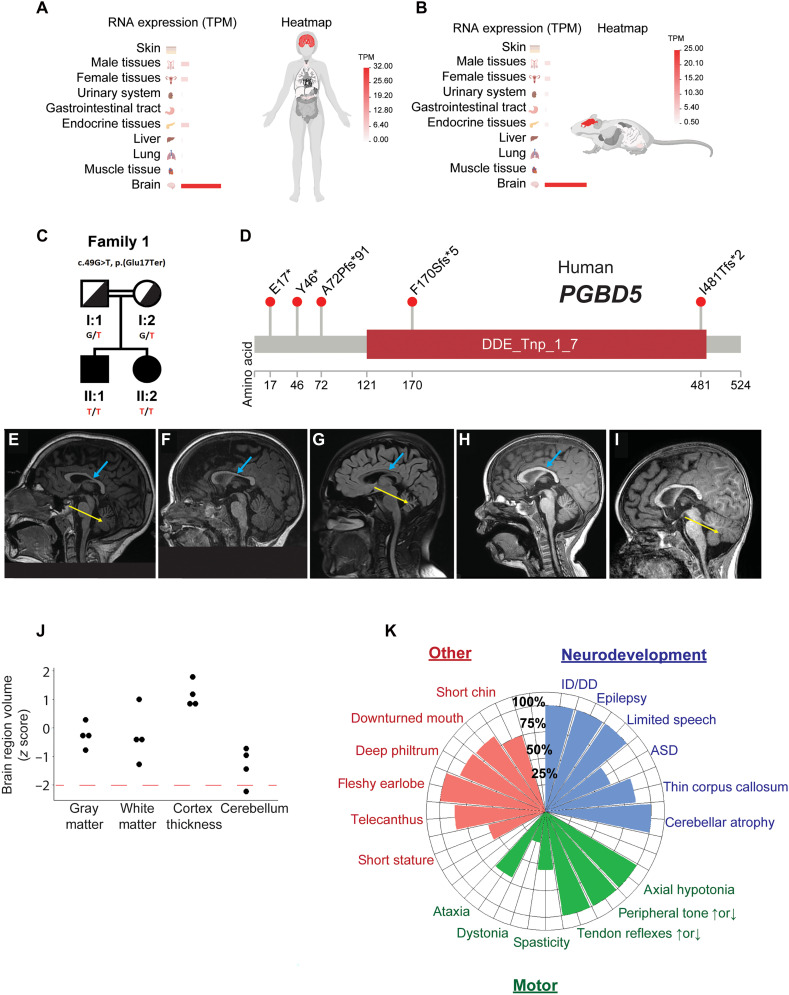
*PGBD5* is specifically expressed in neuronal tissues and its deficiency in humans is associated with abnormal brain development. (**A** and **B**) Bar graphs showing specific neuronal tissue expression of *PGBD5* in human (A) and mouse (B) tissues. Color gradient from red to white indicates gene expression in transcripts per million reads (TPM). (**C**) Pedigree of family 1 demonstrating recessive inheritance of c.49G>T, p.(E17*) genetic variant. Filled symbols indicate individuals affected by PGBD5 deficiency; both parents (I:1, male; I:2, female) are heterozygous (G/T) carriers showing no phenotypic expression, while both children exhibit PGBD5 deficiency phenotypes. Squares represent males; circles represent females. (**D**) Schematic of the primary structure of PGBD5 with observed genetic variants, most of which appear to be loss-of-function upstream of or within the evolutionarily conserved DDE_Tnp_1_7 transposase-homology domain (red box). (**E** to **I**) Sagittal MRI brain images demonstrating thin corpus callosum (thick blue arrow) in patients 2 years and older and decreased cerebellar size (thin yellow arrow) in patients 6 years and older. (E) Patient 1.1 (10 years) with progressive cerebellar atrophy determined after repeat imaging as compared to 4 years of age, (F) patient 1.2 (2 years) with corpus collosum thinning present, (G) patient 2.1 (15 years) with marked cerebellar atrophy, (H) patient 5.1 (3 years) with some thinning of CC, and (I) patient 5.2 (21 months) with cerebellar atrophy. (**J**) Comparison of volumes of different brain structures between four patients with PGBD5 and age-/sex-matched controls. Dashed red line indicates 2 SDs from controls. (**K**) Phenogram summarizing frequency of conserved features in neurodevelopmental, motor, and congenital anomaly domains. Frequency calculated using patients with data provided, excluding N/A from denominator. ID/DD, intellectual disability/developmental delay; ASD, autism spectrum disorder.

First, we explored the developmental expression of PGBD5 and found that PGBD5 is expressed predominantly in nervous system tissues, and the brain in particular (Fig. 1, A and B, and fig. S1, A and B), with the highest expression in glutamatergic neurons, followed by GABAergic neurons, both in humans (fig. S2, A and C) and mice (fig. S2, B and D). To investigate the function of PGBD5 in human brain development, we identified five unrelated consanguineous families including 10 affected individuals with *PGBD5* genetic variants using GeneMatcher (*29*). Exome sequencing analysis demonstrated distinct homozygous *PGBD5* mutations segregating with the affected family members (Fig. 1C and fig. S3, A to D). We confirmed the observed *PGBD5* mutations using genomic polymerase chain reaction (PCR) and Sanger sequencing (fig. S1C and table S1). *PGBD5* mutations in affected individuals consisted of apparent nonsense and frameshift variants, most of which occurred upstream of the evolutionarily conserved aspartate triad thought to be required for the cellular activity of the *PGBD5* transposase-homology domain in cells (Fig. 1D). Expression of cDNAs encoding the observed *PGBD5* c.49G > T p.(E17*) and c.509del p.(F170Sfs*5) mutations led to substantial reduction of PGBD5 protein in human embryonic kidney (HEK) 293T cells (fig. S1, D and E). While additional studies will be needed to establish the effects of observed mutations on endogenous loci in PGBD5-expressing cells, at least some of the phenotypes of the affected individuals can be attributed to the loss of PGBD5 protein.

Affected children with inherited PGBD5 mutations shared conserved clinical phenotypes across neurodevelopmental and motor domains (Fig. 1, E to K; Supplemental Clinical Summaries; table S2). While brain magnetic resonance imaging (MRI) volumes were not quantitatively reduced (Fig. 1, D to J), visual analysis identified thin corpus callosum (six of seven) and reduced cerebellar size associated with widening of the vermis folia (seven of seven), which became more apparent in patients older than 6 years or on follow-up imaging (Fig. 1, E to I). Neurodevelopmental features included intellectual disability and developmental delay (ID/DD; ten of ten), epilepsy (nine of nine), limited or no speech (nine of nine), autism spectrum disorder, or social delay (ASD; four of six). Prominent motor features included axial hypotonia (nine of nine), increased peripheral tone (seven of nine) or decreased peripheral tone (two of nine), increased tendon reflexes (five of nine), or decreased tendon reflexes (four of nine). Less frequently, we observed spasticity that mainly affected the lower limbs (five of nine), intermittent dystonia (three of ten), and ataxia (seven of ten). Some patients were of short stature (five of nine), although height and head circumference were generally normal. We noted some dysmorphic features, including telecanthus (six of seven), fleshy earlobes (eight of eight), deep philtrum (seven of eight), downturned mouth corners (six of seven), and short chin (six of eight; Fig. 1K, data S1, and table S2). While additional patients will be needed to define the full spectrum of human PGBD5 deficiency syndrome, these findings indicate that PGBD5 mutations are associated with developmental delay, intellectual disability, ataxia-dystonia, and epilepsy.

To investigate the physiological functions of PGBD5 in nervous system development, we used dual recombinase-mediated cassette exchange to engineer *Pgbd5^fl/fl^* mice, in which *Pgbd5* exon 4 is flanked by *loxP* sites (*30*). We bred *Pgbd5^fl/fl^* mice with *EIIa-Cre* mice to generate *Pgbd5^−/−^* mice (fig. S4A), as confirmed by genomic PCR and Sanger sequencing (fig. S4C). In situ hybridization microscopy analysis of the hippocampus, which has some of the highest density of Pgbd5-expressing neurons (fig. S1, A and B, and figs. S2, A to D, and fig. S4B), revealed no measurable Pgbd5 exon 4 transcript expression in *Pgbd5^−/−^* mice (fig. S4B), although RNA sequencing analysis revealed residual (~10-fold reduced) *Pgbd5* transcripts lacking exon 4, consistent with potential retention of truncated alleles lacking the transposase-homology domain (fig. S4D). We also engineered *Pgbd5^3xFLAG-HA-P2A-eGFP^* knock-in mice, permitting specific detection of endogenous Pgbd5 expression in cells (fig. S5A). We confirmed that Pgbd5 is expressed in neurons, but not in astrocytes or microglia, by specific costaining with NeuN, glial fibrillary acidic protein (GFAP), and TMEM119, respectively (fig. S5, B to D).

**Fig. 2.**
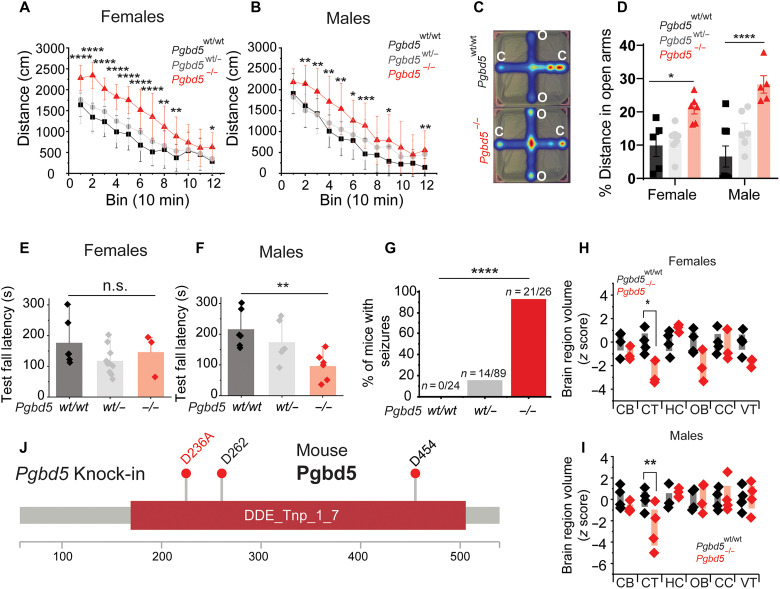
*Pgbd5* knock-out mice reproduce behavioral and brain developmental deficits associated with human *PGBD5* mutation. (**A** and **B**), Distance traveled in locomotor assays of 12-week-old female (A) and male (B) *Pgbd5^wt/wt^* (black), *Pgbd5^wt/−^* (gray), and *Pgbd5^−/−^* (red) mice, demonstrating increased activity of *Pgbd5*-deficient mice (two-way ANOVA *P* = 5.41 × 10^−7^ and *n* = 12 and *P* = 5.47 × 10^−5^, respectively; post hoc Tukey test *P < 0.05, **P < 0.01, ***P < 0.001, ****P < 0.0001; *n* = 12). (**C**) Representative heatmaps of EPM assay (color index from dark blue to red indicates increased time spent in the area), with (**D**) bar plot of the percentage of the distance traveled in the open arm by 12-week-old *Pgbd5^wt/wt^* (black), *Pgbd5^wt/−^* (gray), and *Pgbd5^−/−^* (red) mice. *Pgbd5*-deficient mice exhibit increased propensity to explore open arms (two-way ANOVA *P* = 2.6 × 10^−6^ for genotype and *P =* 0.3 for sex; * Tukey test *P* = 0.019 and ****P = 2.67 × 10^−7^). (**E** and **F**) Rotarod fall latency in females (E) and males (F), showing reduction by *Pgbd5^−/−^* (red) as compared to *Pgbd5^wt/wt^* in male mice (** = ANOVA *P* = 9.2 × 10^−3^, Tukey’s test *P* = 7.5 × 10^−3^ in males; n.s. = ANOVA *P* = 0.2, Tukey’s test *P* = 0.76 ** ANOVA. (**G**) Bar plot of seizure activity of *Pgbd5^−/−^* versus *Pgbd5^wt/wt^* littermates (**** χ^2^ test *P* = 5.8 × 10^−7^). (**H** and **I**) Box plots of *z* scores of brain MRI volumetric measurements of 60-day-old *Pgbd5^−/−^* as compared to *Pgbd5^wt/wt^* mice, showing significant reduction in cortex size in *Pgbd5*-deficient female [(H) * = two-way ANOVA *P =* 9 × 10^−4^, cortex Bonferroni-adjusted *P =* 5.7 × 10^−3^] and male [(I) ** = two-way ANOVA *P =* 0.28, cortex Bonferroni-adjusted *P =* 1.9 × 10^−2^]. (**J**) Pgbd5 primary protein sequence indicating conserved aspartates of mouse Pgbd5 required for genome remodeling activity in cells, corresponding to residues D237, D263, and D455 in human PGBD5 (*21*–*23*). Mouse Pgbd5 exon 2 D236A (ki) substitution is marked in red, corresponding to the mouse ENSMUST00000140012.8 Pgbd5 transcript. O, open arm; C, closed arm; CB, cerebellum; CT, cortex; HC, hippocampus; OB, olfactory bulb; CC, corpus callosum; VT, ventricles.

We found that both *Pgbd5^−/−^* and their *Pgbd5^wt/−^* littermate mice were born at the expected Mendelian ratios (fig. S6, A and B), but *Pgbd5^−/−^* mice were runted and had significantly smaller brains than their wild-type (WT) littermates (*t* test *P* = 3.4 × 10^−3^ and 0.016, for females and males, respectively; fig. S6, C to H). Given the neurodevelopmental deficits associated with PGBD5 deficiency in humans, we performed specific behavioral tests in *Pgbd5^−/−^* mice that correlated with features of human PGBD5 deficiency (fig. S7, A to C). Automated video tracking locomotor test analysis revealed significantly increased locomotor activity of *Pgbd5*^−/−^ and *Pgbd5^wt/−^* mice, compared to their WT littermates [analysis of variance (ANOVA) *P* = 5.4 × 10^−7^ and 5.5 × 10^−7^, for females and males, respectively; Fig. 2, A and B]. We assessed anxiety-like behaviors using the elevated plus maze test (EPM; Fig. 2C). Both female and male *Pgbd5*^−/−^ mice traveled longer distances in the open maze arms (normalized to total distance traveled) compared to their WT littermates, indicating reduced avoidance of the anxiogenic open arm of the EPM, consistent with reduced anxiety-like behavior (ANOVA *P* = 2.7 × 10^−6^; Fig. 2D). Reduced avoidance of female and male *Pgbd5*^−/−^ mice was also reflected by their increased entry and time spent in the open arms (fig. S7, A and B). *Pgbd5^wt/−^* females and males exhibited an intermediate phenotype in open arm time and entries, indicating that a partial deficit in *Pgbd5* expression is sufficient to elicit the EPM phenotype.

Prompted by the motor deficits of *PGBD5*-deficient humans, we assayed *Pgbd5*-deficient mice using the rotarod performance test for motor coordination and balance (Fig. 2, E and F, and fig. S7, D to J). Despite having no significant differences in grip strength (*P* = 0.08; fig. S7H), *Pgbd5^−/−^* male mice exhibited significantly reduced rotarod fall latency, consistent with impaired motor coordination (ANOVA *P* = 9.2 × 10^−3^, Tukey’s test *P* = 0.5 × 10^−3^; Fig. 2, E and F). Both male and female *Pgbd5*^−/−^ mice also exhibited thermal hypersensitivity (fig. S7K), without any apparent gait effects (fig. S7, I and J). Last, we assayed *Pgbd5*-deficient mice for susceptibility to seizures. We found that most *Pgbd5^−/−^* mice developed partial motor and generalized tonic-clonic seizures in response to stressful handling compared to their WT littermates (χ^2^ test *P* = 5.8 × 10^−7^; Fig. 2G). To investigate the anatomical basis of this complex behavioral syndrome, we used high-resolution manganese-enhanced MRI to analyze brain architecture in *Pgbd5*-deficient mice. This imaging revealed significant reductions of the cortical volumes in *Pgbd5^−/−^* male and female mice, assayed using quantitative volumetric mouse brain atlas analysis (ANOVA Bonferroni-adjusted *P =* 1.9 × 10^−2^ and 9 × 10^−4^, respectively; Fig. 2, H and I, and fig. S8). Overall, *Pgbd5*-deficient mice display complex behavioral deficits, including seizures, behavioral and motor deficits, and structural brain abnormalities that resemble the human PGBD5–deficiency disorder.

**Fig. 3.**
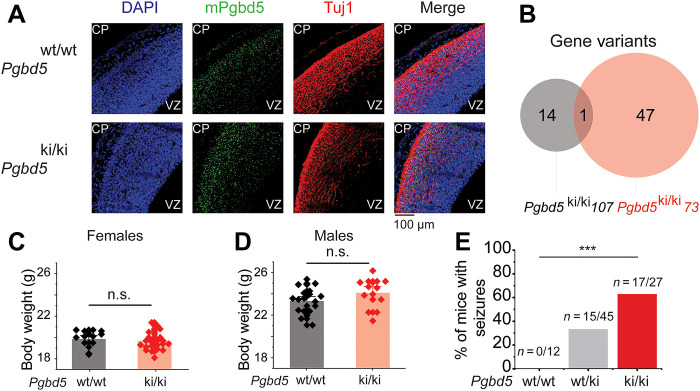
*Pgbd5* knockin mice reproduce behavioral and brain developmental deficits of *Pgbd5* knockout mice. (**A**) Representative fluorescence in situ hybridization micrographs of coronal sections of heads of 14.5-day-old *Pgbd5^ki/ki^* as compared to *Pgbd5^wt/wt^* littermate embryos, showing similar expression of *Pgbd5* transcripts between *Pgbd5^wt/wt^* and *Pgbd5^ki/ki^* littermates. Green staining indicates mouse *Pgbd5* RNA, red indicates Tuj1 staining of postmitotic neurons, and blue denotes nuclei stained with 4′,6-diamidino-2-phenylindole (DAPI). Scale bar, 100 μm. CP, cortical plate; VZ, ventricular zone. (**B**) Venn diagram of gene variants detected in the WGS of *Pgbd5^ki/ki^ 107* and *Pgbd5^ki/ki^73* founder lines. Only the targeted *Pgbd5^ki^* gene variant was shared between founder lines. (**C** and **D**) Total body weight of 60-day-old *Pgbd5^wt/wt^* (black), and *Pgbd5^ki/ki^*(red) mice, shows no difference of total weights in female [(C) n.s. *P =* 0.3] and male [(D) n.s. *P =* 0.6] mice. (**E**) Bar plot of seizure activity of *Pgbd5^−/−^* versus *Pgbd5^ki/ki^* littermate mice (*** χ^2^ test *P* = 7.4 × 10^−05^). *n* indicates number of mice with seizures over total number of mice assayed.

PiggyBac-type enzymes use conserved aspartate residues to catalyze DNA hydrolysis and rearrangements, with divergent evolutionarily conserved aspartate triad required for the DNA remodeling activities of PGBD5 in cells, albeit without current evidence of direct nuclease activity in vitro (*21*, *22*). Whether PGBD5 functions enzymatically as a transposase, recombinase, a different type of nuclease or involves another nuclease entirely needs to be determined (*25*). To test whether mouse brain development requires specific Pgbd5 genome remodeling activity, we used CRISPR engineering to generate *Pgbd5^D236A;D236A^* knock-in mice (*Pgbd5^ki/ki^*; Fig. 2J and fig. S9A), in which one of the vertebrate evolutionarily conserved aspartate residues required for cellular DNA activity was mutated to inactive alanine (*21*). We confirmed that the analogous mutation in human *PGBD5* does not impair cellular protein stability by Western immunoblotting or its ability to associate with chromatin in human cells by chromatin immunoprecipitation sequencing (*21*, *22*). We verified the *Pgbd5^D236A^* mutation in two independent founder strains using genomic PCR and Sanger sequencing (fig. S9A), germline transmission by restriction enzyme mapping (fig. S9B), and lack of off-target gene mutations by whole-genome sequencing (WGS) (Fig. 3B and table S3). Homozygous *Pgbd5^ki/ki^* mice exhibited unaltered expression of endogenous Pgbd5 mRNA compared to their *Pgbd5^wt/wt^* littermates, as assessed using in situ hybridization with *Pgbd5*-specific probes (Fig. 3A). *Pgbd5^ki/ki^* mice showed no difference in body weights as compared to their WT littermates (Fig. 3, C and D) but exhibited tonic-clonic seizures similar to *Pgbd5*-deficient mice (χ^2^ test *P* = 7.4 × 10^−05^; Fig. 3E). Thus, brain functions of Pgbd5, at least in part, require specific aspartate functions of its transposase-homology domain, although it remains to be determined whether this involves direct PGBD5 nuclease activity or allosteric effects on a cofactor nuclease.

**Fig. 4.**
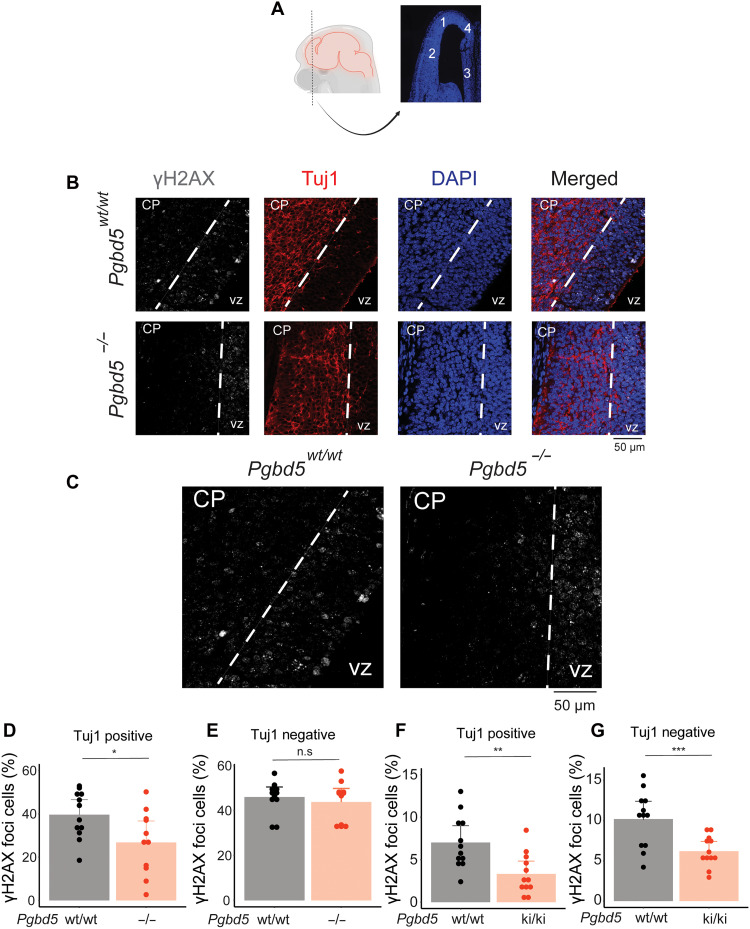
*Pgbd5* is required for developmental induction of DNA breaks in postmitotic cortical neurons. (**A**) Schematic showing a representative coronal section of a 14.5-day-old embryo mouse forebrain and the regions selected for further quantification. (**B**) Representative immunofluorescence micrographs of *Pgbd5^wt/wt^* and *Pgbd5^−/−^* 14.5-day-old littermate embryos. DAPI/Hoescht-33342 shown in blue stains nuclei, γH2AX in white indicates sites of double-strand DNA break repair, and Tuj1 in red marks differentiated postmitotic neurons; CP, cortical plate; VZ, ventricular zone. (**C**) Enlarged representative γH2AX immunofluorescence micrographs from (A) of *Pgbd5^wt/wt^* (left) and *Pgbd5^−/−^* (right) 14.5-day-old littermate embryos stained for γH2AX in white. (**D** to **G**) Quantification of γH2AX in postmitotic (Tuj1 positive) and proliferating neurons (Tuj1 negative) in the *Pgbd5^wt/wt^* and *Pgbd5^ki/ki^* mice. Tuj1-positive and -negative neurons were identified by demarcating image regions where Tuj1 signal was present and absent, respectively. [(D) and (E)] Bar plots showing the percentages of cells with punctate γH2AX staining in Tuj1-positive (D) and Tuj1-negative neuronal regions (E) in *Pgbd5^wt/wt^* versus *Pgbd5^−/−^* mice (*t* test *P = 0.029 and n.s. *P =* 0.6 for % of positive cells). [(F) and (G)] Bar plots showing the percentages of cells with punctate γH2AX staining in Tuj1-positive (F) and Tuj1-negative neuronal populations (G) in *Pgbd5^wt/wt^* versus *Pgbd5^ki/ki^* mice (*t* test **P = 3.8 × 10^−3^ and ***P = 2.9 × 10^−3^ for Tuj1-positive and -negative neuronal populations, respectively). Note that while both *Pgbd5^−/−^* and *Pgbd5^ki/ki^* mice are derived from the B6 mouse strain, they exhibit apparent differences in the basal levels of γH2AX signals.

Mammalian neurogenesis occurs largely during embryonic development, with mouse cortical development peaking in the mid-to-late embryonic stages (*31*). Given the well-defined layered organization of the mouse motor cortex, we analyzed its architecture in 14.5-day-old [embryonic day 14.5 (E14.5)] embryos. Prior studies of this developmental period have also documented that postmitotic neurons accumulate extensive DNA breaks and activate end-joining DNA repair as they migrate to the mantle layer upon differentiation of progenitor neuroblasts in the ventricular zone (*2*, *3*, *7*). Thus, we used a neuron-specific tubulin isoform Tuj1 as a specific marker of postmitotic neurons (*32*) and immunofluorescence microscopy to examine postmitotic neurons in the brain cortices of 14.5-day-old embryonal (E14.5) *Pgbd5^−/−^* mice (Fig. 4, A to C, and figs. S10 to S14).

Using γH2AX as a specific surrogate of neuronal DNA break repair (*33*), we observed a significant reduction in the number of neurons with γH2AX foci specifically among postmitotic (Tuj1-positive), compared to proliferating (Tuj1-negative), neuronal precursors in *Pgbd5^−/−^* mice compared to their WT littermates (*t* test *P* = 0.029; Fig. 4, D and E, and fig. S15, A and B). We confirmed the specificity of this effect by analyzing the fraction of Tuj1-negative neurons in brain cortical neurons, which showed no significant differences between *Pgbd5*-deficient and WT littermate brains (Fig. 4, D and E, and fig. S16A). The observed Pgbd5-dependent neuronal γH2AX foci were specifically induced during cortical neuronal development in E14.5 embryos, as analysis of E12.5 brain cortices, which contain only a single mantle layer, revealed no significant differences (fig. S17). *Pgbd5^ki/ki^* mice also exhibited significant reduction of γH2AX foci as compared to WT littermate controls (*t* test *P* = 3.8 × 10^−3^ and 2.9 × 10^−3^ for Tuj1-positive and negative neurons, respectively; Fig. 4, F and G). Thus, Pgbd5 and the cellular genome remodeling activity of its transposase-homology domain are specifically required for the developmental induction of DNA breaks and/or their resolution during cortical brain development.

**Fig. 5.**
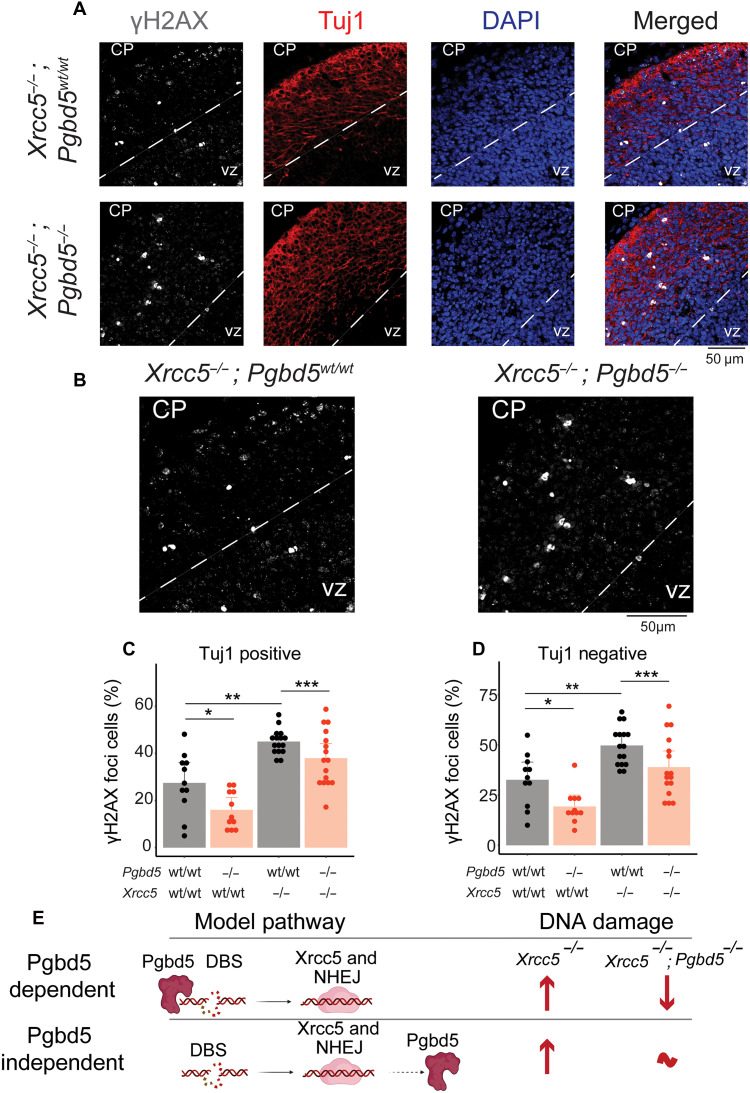
*Xrcc5* is required for *Pgbd5*-induced double-strand DNA break repair. (**A**) Representative immunofluorescence micrographs of *Xrcc5^−/−^;Pgbd5^wt/wt^* (top) and *Xrcc5^−/−^*;*Pgbd5^−/−^* (bottom) 14.5-day-old littermate embryos stained for γH2AX. DAPI nuclear staining is shown in blue; γH2AX indicates sites of double-strand break repair (white), and Tuj1 marks postmitotic neurons (red); CP, cortical plate; VZ, ventricular zone. (**B**) Enlarged representative immunofluorescence micrographs of *Xrcc5^−/−^*;*Pgbd5^wt/wt^* and *Xrcc5^−/−^*;*Pgbd5^−/−^* 14.5-day-old litter mate embryos from (A). (**C** and **D**) Quantification of nuclear γH2AX in postmitotic neurons (Tuj1 positive) and proliferating neurons (Tuj1 negative). Tuj1-positive and -negative neurons were identified by demarcating image regions where Tuj1 signal was present and absent, respectively. Bar plots showing percentages of cells with punctate γH2AX staining in Tuj1-positive [(C) two-way ANOVA Dunn-Šidák adjusted *P = 7.6 × 10^−2^, **P = 4.2 × 10^−3^, and ***P = 8.6 × 10^−2^], and Tuj1-negative neurons [(D) *P = 5.0 × 10^−2^ and **P = 2.0 × 10^−4^, ***P = 0.25 for postmitotic and proliferating neurons, respectively]. (**E**) Schematic showing potential genetic interaction models between *Pgbd5* and *Xrcc5* in cortical neuronal developmental DNA break repair to discriminate between Pgbd5-dependent and Pgbd5-independent developmental DNA breaks. DSB, double-strand DNA break; NHEJ, nonhomologous end joining repair. Arrows denote relative levels of DNA damage in respective mouse genotypes.

While Pgbd5 is a vertebrate evolutionarily conserved and transposase-derived gene with cellular genome remodeling activities (*21*, *22*), it is possible that the observed DNA breaks in neurons occur independently of its cellular DNA breakage activity. To determine whether Pgbd5-dependent neuronal DNA breaks require DNA double-strand break repair, we analyzed the genetic interaction between *Pgbd5* and *Xrcc5/Ku80*, the key factor in nonhomologous end joining (NHEJ) DNA repair (Fig. 5). NHEJ DNA repair is required for the ligation of double-strand DNA breaks induced by many “cut-and-paste” DNA transposase enzymes and their domesticated derivatives like PGBD5 and RAG1/2 (*10*, *34*). Similar to human DNA damage repair deficiency syndromes, such as AT and Seckel syndromes, *Xrcc5^−/−^* mice have neurodevelopmental defects, associated with unrepaired DNA breaks and extensive neuronal apoptosis during cortical development, as well as severe combined immunodeficiency due to the failure to repair RAG1/2-induced DNA breaks and rearrangements in developing lymphocytes (*35*).

First, we confirmed that *Xrcc5^−/−^* mice failed to produce normal T and B lymphocytes, as assayed by fluorescence-activated cell scanning using CD4/CD8 and B220/immunoglobulin M (IgM)–specific antibodies of thymus and spleen, respectively, as compared to their WT or *Pgbd5^−/−^* littermates (figs. S22, I and J, and S23). In agreement with prior studies, *Xrcc5^−/−^* mice showed a significant increase in the number of neurons with γH2AX breaks as compared to their WT littermates in postmitotic Tuj1-positive neurons (two-way ANOVA Dunn-Šidák adjusted *P* = 4.2 × 10^−3^; Fig. 5C) and proliferative Tuj1-negative progenitors (*P* = 2.0 × 10^−4^; Fig. 5D). We found that *Pgbd5^−/−^*;*Xrcc5^−/−^* mice had significantly less DNA damage, as compared to their *Pgbd5^wt/wt^*;*Xrcc5^−/−^* littermates, preferentially in postmitotic Tuj1-positive neurons (two-way ANOVA Dunn-Šidák adjusted *P* = 8.6 × 10^−2^ and 0.25 for Tuj1-positive and negative neurons, respectively; Fig. 5, C and D, and figs. S15, C and D, and S18 to S20). We further confirmed the statistical significance of this effect using a generalized linear model that accounted for potential dependencies among different populations of neurons quantified across cortical regions (Tuj1-positive *Pgbd5^wt/wt^*;*Xrcc5^−/−^* versus *Pgbd5^−/−^*;*Xrcc5^−/−^* Wald-test *P* = 3.7 × 10^−2^, although *Pgbd5^wt/wt^*;*Xrcc5^wt/wt^* versus *Pgbd5^−/−^*;*Xrcc5^wt/wt^* exhibited Wald-test *P* = 3.7 × 10^−1^, suggesting that Xrcc5 deficiency unmasks Pgbd5 effects). Thus, Pgbd5-induced neuronal DNA damage repair requires Xrcc5, preferentially in postmitotic cortical neurons (Fig. 5E).

Commensurate with the physiological function of developmental neuronal DNA break repair, we found that *Pgbd5^−/−^*;*Xrcc5^−/−^* mice were similarly runted and failed to thrive as compared to their *Pgbd5^wt/wt^*;*Xrcc5^−/−^* littermates (figs. S22, A to H, and S23). These features were associated with increased neuronal cell death, as measured by terminal deoxynucleotidyl transferase–mediated deoxyuridine triphosphate nick end labeling (TUNEL) specifically in E14.5, but not E12.5, brains (*t* test *P* = 1.5 × 10^−2^ and *P =* 0.89, respectively; fig. S24). In all, these findings indicate that Pgbd5 is required for the developmental induction of neuronal DNA breaks and cortical brain development.

To determine whether Pgbd5 induces somatic DNA rearrangements during brain development, we used PCR-free paired-end Illumina WGS of diverse anatomically dissected brain regions from multiple individual *Pgbd5*-deficient and WT littermate mice. Current single-cell DNA sequencing methods enable accurate detection of single nucleotide variation, but their requirements for DNA amplification prevent the detection of larger rearrangements, such as those expected from DNA nucleases (*36*). Bulk PCR-free DNA sequencing is not sufficiently sensitive to detect DNA rearrangements occurring in single neurons. However, we reasoned that if Pgbd5 promotes somatic neuronal DNA rearrangements, its developmental activity would yield recurrent somatic signals in multiple diverse WT, but not *Pgbd5^−/−^* littermate, brains via involvement of shared loci and/or sequences in bulk cell sequencing.

**Fig. 6.**
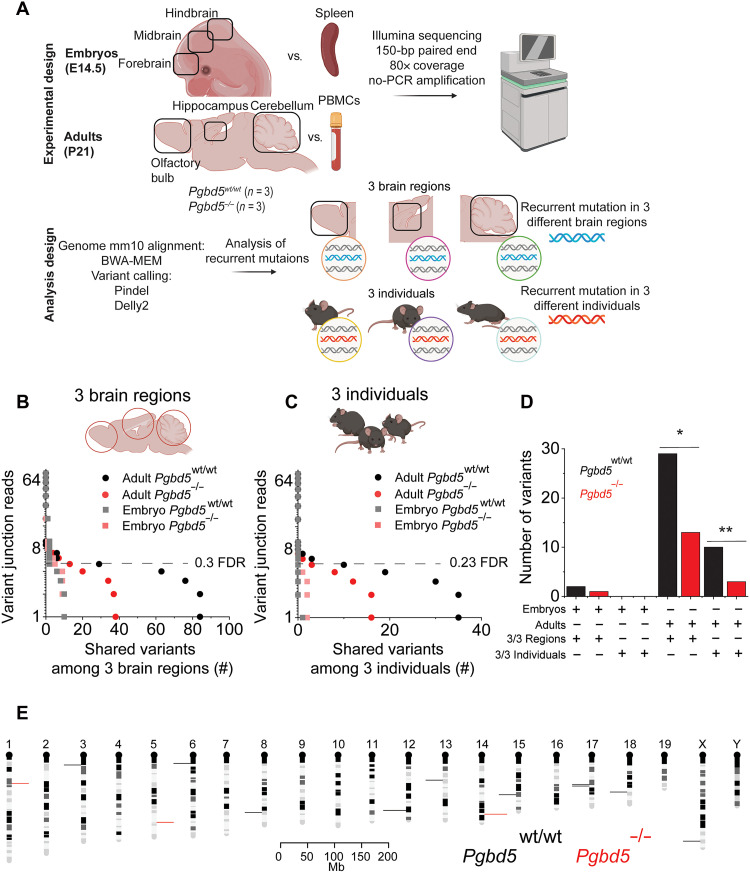
*Pgbd5* is required for recurrent somatic DNA rearrangements in developing mouse brain. (**A**) Schematics for somatic WGS analysis of neuronal and nonneuronal tissues from three *Pgbd5^wt/wt^* and three *Pgbd5^−/−^* adult and embryonal littermate mice. (**B** to **D**) Dot plots showing numbers of somatic structural variants at different variant junction read thresholds shared across cerebellum, hippocampus, and olfactory bulb brain regions (B) and three individuals (C) in adult and embryonal *Pgbd5^wt/wt^* (black circles and gray squares, respectively) and *Pgbd5^−/−^* (red circles and light-red squares, respectively). The overlap among structural variants was calculated using the break point analysis (fig. S27D): 5′ and 3′ DNA break point ± 350 bp requiring an overlap of at least 1%. There are significantly more recurrent somatic structural variants in *Pgbd5^wt/wt^* with support of at least five variant junction reads in the recurrent events shared among three individuals and three brain regions (*χ^2^ test *P* = 2.2 × 10^−145^ and 2.8 × 10^−133^, respectively). (D) Bar plot summarizing the results from (B) and (C) using the support threshold of at least five variant junction reads. Significant differences between the number of recurrent somatic DNA rearrangements between adult *Pgbd5^wt/wt^* and *Pgbd5^−/−^* shared among three individuals and three brain regions (**χ^2^ test *P* = 1.6 × 10^−17^ and 1.3 × 10^−9^, respectively). (**E**) Mouse chromosome ideograms showing the locations of recurrent somatic DNA rearrangements in three individuals observed in *Pgbd5^wt/wt^* (black) and *Pgbd5^−/−^* (red) brains; bin = 1 million bases.

We tested this conjecture by analyzing somatic DNA rearrangements observed using PCR-free paired-end Illumina WGS analysis of peripheral blood mononuclear cells (PBMCs) isolated from 21-day-old mice (mean genome coverage 90-fold; Fig. 6A). First, we validated that this analysis was not biased by sequencing coverage (fig. S25, A and B) and produced accurate detection of somatic DNA variants based on the allele frequencies in matched tissues (fig. S25, C to F). We analyzed the resultant sequencing data using recently developed methods optimized for the accurate detection of somatic genome variation (*37*–*39*). Consistent with the known somatic V(D)J DNA recombination activity of RAG1/2 in blood lymphocytes, we observed somatic deletions of the *Igkj1* and *Igkj2* loci (among other immunoglobulin receptor genes) with common break points in multiple sequencing reads in PBMCs as compared to matched brain tissue (mean variant fraction = 0.015; fig. S26A). Lack of apparent somatic deletions of *Igkj1* and *Igkj2* or related immunoglobulin gene loci in fetal spleens of 14.5-day-old mouse embryos, as compared to their matched brain tissue, confirmed the specificity of this approach (fig. S26A), consistent with the known absence of RAG1/2 activity in fetal hematopoietic cells in mouse spleen (*40*). In contrast, we observed clonal deletions of *Pgbd5* exon 4 in both adult PBMCs and fetal spleens of *Pgbd5^−/−^* mice, but not in their WT littermates (fig. S26B).

Using this comparative approach to detect developmental somatic DNA rearrangements by the domesticated RAG1/2 DNA recombinase in blood cells, we examined somatic genomic variation of brain tissues dissected from *Pgbd5^wt/wt^* and *Pgbd5^−/−^* littermate mice. We performed independent analyses to quantify somatic single-nucleotide variants (SNVs) and DNA rearrangements, such as deletions, and then tested for their recurrence by comparing genomic locations of somatic variants in individual mice or their anatomic brain regions (see Materials and Methods). We observed no significant differences in somatic SNVs in the brain tissues of both 30-day-old adult and 14.5-day-old embryonal *Pgbd5^wt/wt^* as compared to their *Pgbd5^−/−^* littermate mice, consistent with their equal chronological and biological age (median allele fraction = 0.096 and 0.094 for adult *Pgbd5^wt/wt^* and *Pgbd5^−/−^* mice, respectively; fig. S28, A and B).

In agreement with the stochastic nature of somatic nucleotide substitutions, most of which are due to DNA replication in proliferating tissues (*41*), we also found no genomic regions that recurrently accumulated somatic SNVs across different brain regions or different individual mice, which have an apparently random distribution across the mouse genome (fig. S27, A and B, and table S4). We then focused on the analysis of somatic deletions, insertions, and duplications in adult and embryonal *Pgbd5^wt/wt^* brains, as compared to their *Pgbd5^−/−^* littermate controls (fig. S28). While we observed some differences in the various types of structural DNA rearrangements, there were no statistically significant differences in the total numbers of somatic DNA rearrangements between *Pgbd5^wt/wt^* and *Pgbd5^−/−^* brains, both in adults and embryos (fig. S28).

In contrast, individual adult *Pgbd5^wt/wt^* mice showed significantly more recurrent somatic DNA rearrangements, both among different individual mice and their cerebella, hippocampi, and olfactory bulbs, as compared to their *Pgbd5^−/−^* littermates. This was detected using both recurrence of somatic structural rearrangement break points and their complete overlaps (24 versus 12, and 10 versus 2, respectively; χ^2^ test *P* = 1.3 × 10^−9^ and 1.6 × 10^−17^, respectively; Fig. 5, A to D, and fig. S27, C to G). There were no significant differences in the recurrence of somatic DNA rearrangements in 14.5-day-old embryonal brain tissues isolated from *Pgbd5^wt/wt^* and *Pgbd5^−/−^* littermate embryos (Fig. 6D and fig. S27G), consistent with the onset of Pgbd5/Xrcc5-dependent DNA break repair during this developmental period.

Last, recurrent somatic DNA rearrangements shared across individual mice and brain regions showed specific genomic distributions (Fig. 5E, fig. S27H, and table S4). Manual inspection of sequencing reads of a subset of DNA rearrangements was consistent with their somatic induction in brain tissues in *Pgbd5^wt/wt^* but not *Pgbd5^−/−^* littermate mice (fig. S29, A to C, and table S4). While the definition of physiological Pgbd5 genomic targets and their rearranged sequences will require the development of improved single-cell genomic sequencing methods, we propose that the somatically rearranged genomic elements identified here represent signals of developmental physiological Pgbd5 activity in normal brain development.

**Fig. 7.**
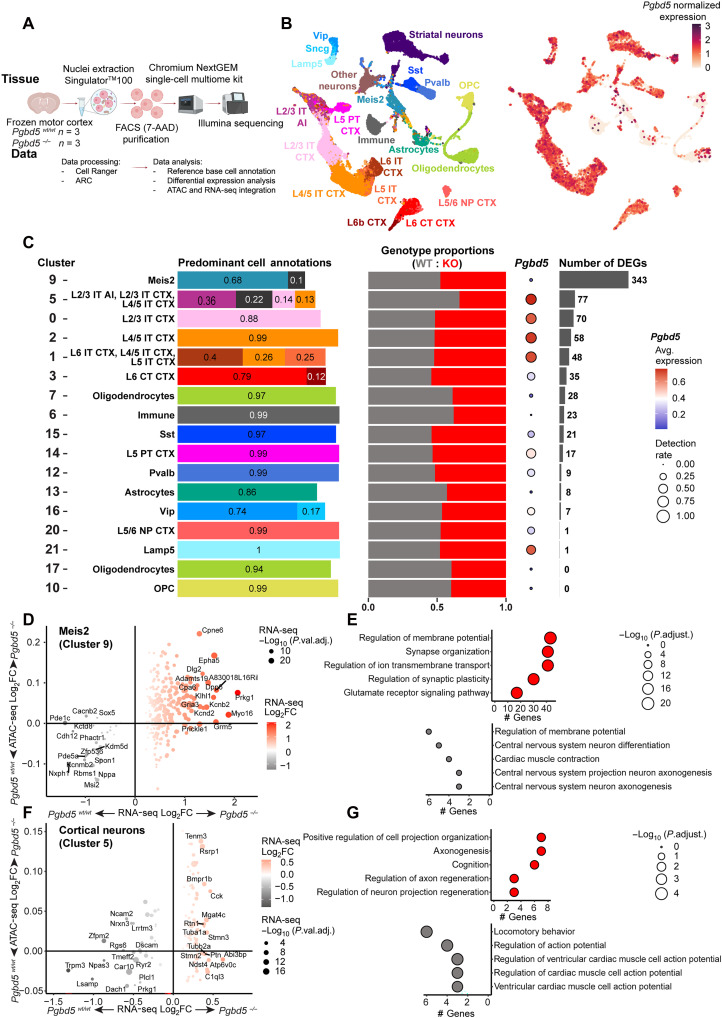
*Pgbd5* deficiency alters gene expression in distinct cortical neurons. (**A**) Schematic of experimental procedures for analysis of combined single-nucleus RNA and ATAC sequencing of brain motor cortices from three *Pgbd5^wt/wt^* and three *Pgbd5^−/−^* littermate mice. (**B**) Uniform Manifold Approximation and Projection (UMAP) plots of single nuclei gene expression from brain motor cortices of *Pgbd5^wt/wt^* and *Pgbd5^−/−^* littermates, colored by their classification with respect to the reference atlas of normal mouse brain cortex (left; *n* = 18,107 and 14,359, respectively). Right UMAP is colored by *Pgbd5* expression (normalized white to dark red). (**C**) Cell clusters with greater than 200 nuclei corresponding to cell populations of cortical origin in *Pgbd5^wt/wt^* (gray) and *Pgbd5^−/−^* (red) mice, excluding striatal neurons and other neurons. From left to right: proportions of predominant cell type annotations per cluster; proportion of each genotype per cluster; expression (dot color) and detection rate (dot size) of *Pgbd5* expression in WT cells of each cluster; number of DEGs (log_2_FC > 0.25, adjusted *P* < 0.05) between the genotypes per cluster. (**D** to **G**) Differential expression and promoter accessibility analysis in clusters corresponding to Meis2 cluster 9 [(D) and (E)] and cortical cluster 5 neurons [(F) and (G)]. Bubble plots showing changes in gene expression correlated with changes in chromatin accessibility at the corresponding gene promoter regions (±2.5 kb from TSS). Only genes with significant changes in expression (adjusted *P* < 0.05) are plotted in Meis2 cluster 9 (D) and cortical intratelencephalic (IT) neurons (F). Top GO pathways ranked by the number of genes showing adjusted *P* values in bubble size for DEGs up-regulated in knockout (top) and WT (bottom) cells of the corresponding cluster in Meis2 cluster 9 (E) and cortical IT cluster 5 neurons (G).

To identify specific neuronal populations that require Pgbd5 activity during brain development, we performed single-nucleus RNA sequencing combined with transposase-accessible chromatin sequencing (ATAC) of nuclei isolated from the motor cortex of three 21-day-old *Pgbd5^wt/wt^* and three *Pgbd5^−/−^* littermate mice (Fig. 7A and table S5). Upon mapping the observed gene expression onto the developmental ontogeny of normal mouse cortex using two atlases (*42*, *43*), we clustered the gene expression states of detected nuclei. This strategy identified specific Pgbd5-expressing neuronal populations, as compared to astrocytes, oligodendrocytes, and immune cells, most of which lack Pgbd5 expression (Fig. 6B, fig. S30, and table S6). First, we confirmed that the *Pgbd5^wt/wt^* and *Pgbd5^−/−^* brain cortices had relatively equal sampling, consistent with their overall preserved morphologic organization (fig. S4). We found no significant differences in the proportions of annotated cell types between *Pgbd5^wt/wt^* and *Pgbd5^−/−^* brain cortices (fig. S31).

In contrast, we found significant differences in gene expression of specific populations of neurons with both relatively high and low Pgbd5 expression between *Pgbd5^wt/wt^* and *Pgbd5^−/−^* brains (Fig. 7C). This included the large population of high Pgbd5-expressing intratelencephalic (IT) glutamatergic pyramidal neurons of layers 2/3, 4/5, and 6 that project to other cortical areas, and the striatum (*44*), as well as the much smaller population of low Pgbd5-expressing Meis2 GABAergic interneurons that are present in the cortical white matter and likely represent projection neuron precursors (Fig. 7C) (*45*, *46*). Pyramidal tract neurons, which are the other major cortical pyramidal neurons that project to subcortical structures, as well as cortical GABAergic parvalbumin, somatostatin, Vip interneurons, had relatively few differentially expressed genes (DEGs), consistent with the distinct function of Pgbd5 in specific neuronal populations (Fig. 7C). Thus, loss of Pgbd5 induces distinct changes in the organization and gene expression of cortical neurons.

We next assessed changes in the chromatin accessibility of promoter regions of DEGs between *Pgbd5^wt/wt^* and *Pgbd5^−/−^* cells. Significantly affected cortical neuronal populations included glutamatergic cluster 5 layers 2/3 and 4/5 IT and cluster 9 GABAergic Meis2 neurons (Fig. 6, D to G, and fig. S32). We also observed substantial correlations between differential gene expression and chromatin accessibility of distinct sets of genes (Fig. 6, D and F). The concordance between Pgbd5-dependent gene expression and chromatin accessibility suggests that Pgbd5 deficiency leads to the dysregulation of specific gene expression within neuronal populations. Gene ontology pathway analysis of DEGs from the cortical neuronal populations revealed multiple sets of genes involved in the regulation of neuronal membrane potentials, synapse organization, ion channel signaling, and neuronal and axonal projection regeneration, among other neuronal functions (Fig. 6, E to G, and fig. S31, A and B). At least in part, this may explain the phenotype of human PGBD5 deficiency, including developmental delay, intellectual disability, ataxia-dystonia, and in particular epilepsy, given the importance of an imbalance in excitatory and inhibitory neuronal activity in seizure disorders (*47*). In all, these results indicate that Pgbd5 is required for the function of specific excitatory and inhibitory cortical neurons.

## DISCUSSION

Although somatic genetic mosaicism has been documented extensively in diverse tissues, and somatic genetic diversification during neuronal development was originally proposed more than 50 years ago (*11*), the existence of physiological somatic DNA rearrangements in vertebrate brain development has not been proven so far. Here, we demonstrate that an evolutionarily conserved domesticated DNA transposase–derived PGBD5 causes double-strand DNA breaks in normal neuronal and mammalian brain development. We provide evidence that PGBD5 is required for normal brain development in humans and mice. Its genetic inactivation constitutes the PGBD5 deficiency disorder, characterized by developmental delay, intellectual disability, language and motor impairments, seizures, and reductions in corpus callosum and cerebellar size. While the exact enzymatic mechanism of PGBD5 remains to be determined, studies of mice engineered to express the *Pgbd5^D236A;D236A^* mutant with impaired cellular DNA remodeling activity are consistent with its requirement for physiologic function of PGBD5.

We observe that Pgbd5 is responsible for recurrent somatic DNA breaks in mouse brains, explaining the observations of the requirement of NHEJ DNA repair for mammalian brain development (*48*, *49*). Like RAG1/2-dependent somatic genetic diversification during normal lymphocyte development, mammalian neuronal development also requires the evolutionarily conserved NHEJ DNA repair factor XRCC5/Ku80, which we now show to be associated with PGBD5-dependent neuronal genome rearrangements. While we cannot exclude the formal possibility that PGBD5 induces DNA breaks to promote cortical neuronal death during the same developmental period, we provide evidence that Pgbd5-induced somatic DNA rearrangements affect recurrent neuronal chromosomal loci. In all, this study establishes the human PGBD5 deficiency disorder and identifies distinct neuronal populations and Pgbd5-dependent gene expression programs required for normal mammalian brain development and function. This work also sets a foundation for the identification of molecular mechanisms of PGBD5 and its substrates for neuronal genetic diversification and self-organization in brain development.

PGBD5-mediated somatic neuronal DNA rearrangements may offer a genetic mechanism for neuronal selection and developmental apoptosis, which are known to affect a large subset of cells produced during mammalian neuronal development (*50*–*52*). Many studies have implicated DNA replication as a cause of somatic genetic brain mosaicism. However, this mechanism does not explain how DNA breaks and repair occur in postmitotic neurons. The data presented here offer a plausible mechanism by which physiological somatic DNA rearrangements induced by PGBD5 may contribute to somatic neuronal diversification and cellular selection as progenitor neuroblasts differentiate and exit the cell cycle and migrate from the ventricular zone to the mantle layer, where postmitotic neurons exhibit NHEJ-dependent DNA damage repair and apoptosis (*2*, *3*, *7*). An independent concurrent study by Gustincich and Sanges and colleagues identified Pgbd5 as a cause of developmental neuronal DNA breaks in mice, with Pgbd5 being required for normal brain cortical neuronal migration and differentiation (*53*).

Since RAG1/2 targets distinct genomic loci in developing B and T lymphocytes, PGBD5 targets may also depend on neuronal differentiation and function and, in the case of the brain cortex, the specific neuronal populations identified in this study (Fig. 6, D to G, and figs. S31 and S32). Future studies will be needed to define PGBD5 functions in various brain regions, including the hippocampus and medial temporal regions, given the prominent seizure phenotype of PGBD5 deficiency. It is also possible that PGBD5 has additional nuclease-independent functions in nervous system development, such as those mediated by interactions with chromatin and other cellular factors.

While we favor the conclusion that PGBD5 acts directly on DNA (*21*, *22*, *25*), additional biochemical and structural studies will be needed to define the exact enzymatic mechanisms of PGBD5 cellular activities and their developmental regulatory factors, including the possibility that PGBD5 promotes somatic DNA rearrangements through recruitment of other nucleases and chromatin remodeling factors. Last, we cannot exclude noncentral nervous system contributions to the developmental defects observed in PGBD5-deficient mice and humans, as PGBD5 is likely expressed in other neuronal tissues, such as neuroendocrine cells and the peripheral nervous system.

We must emphasize that PGBD5-dependent DNA rearrangements are not solely responsible for the physiological requirement for DNA damage repair in nervous system function. For example, postmitotic neurons also require XRCC1-dependent base excision/single-strand break repair due to single-strand DNA breaks induced by developmental cytosine demethylation (*50*). Recent studies have also shown that brain aging may involve additional somatic genetic processes (*51*, *52*). Here, we used amplification-free DNA sequencing, which has limitations in specificity and sensitivity. Further studies using amplification-free single-cell analyses will be needed to establish specific somatic neuronal DNA rearrangements and their functions, as recently shown by mapping recurrent mosaic copy number variation in human neurons (*18*).

Developmentally controlled DNA rearrangements have been found in diverse biological processes. For example, in addition to the function of the domesticated DNA transposase RAG1/2 in immunoglobulin receptor gene diversification in vertebrate lymphocytes (*54*), the Spo11 DNA recombinase initiates recombination in eukaryotic meiosis (*55*), the Kat1 DNA transposase controls the yeast mating switching (*56*), and the PiggyMac DNA transposase mediates somatic DNA elimination during macronucleus development in ciliates (*57*). PGBD5-dependent mammalian neuronal genome rearrangements suggest that other evolutionarily conserved DNA transposases may be domesticated as developmental somatic genetic remodelers. This would provide molecular mechanisms for genetic diversification during physiological somatic tissue and organ development. In turn, dysregulation of these processes can cause deleterious somatic genetic mutations, leading to disease. In the case of RAG1/2 and PGBD5, their dysregulation causes somatic oncogenic DNA rearrangements in blood cancers and solid tumors affecting children and young adults (*58*). Thus, dysregulation of PGBD5 functions during brain development may also contribute to the somatic DNA rearrangements in specific neurodevelopmental disorders.

## MATERIALS AND METHODS

### Patient recruitment, sequencing, and assessment

Whole-exome sequencing (WES) was performed first on family 2 using whole-exome Illumina sequencing with mean depth of coverage of 40×. A homozygous 1–base pair (bp) deletion resulting in a frameshift in PGBD5 was identified chr1:230492889 (hg19) NM_001258311.2 c.509 GA>G p.(Phe170SerfsTer5). Four additional families were identified through GeneMatcher (*25*). Family 1 had a homozygous nonsense variant identified via WES as chr1:230425880C>A (hg38) NM_001258311.1 c.49G>T p.(Glu17*). Family 3 WES performed by Centogene identified chr1:230425791G>T (NM_001258311.2)c.138C>A (p.Phe170Serfs*5). Family 4 WES performed at UCL Queen Square Genomics identified as chr1:230323510del49 (NM_001258311.2)c.1442_1490del(p.Ile481ThrfsTer2). All variants had gnomAD frequency = 0. Variants for families 1, 2, and 4 were additionally verified by Sanger sequencing (fig. S1C).

Family 1 Sanger sequencing was performed by Integragen on genomic DNA isolated from peripheral blood using forward primer 5′ GCTGGGAGGCACTGTGG and reverse primer 5′ CAGCCCACGGAGAGTCTG. Family 2 Sanger sequencing was performed by Functional Bio on DNA isolated from peripheral blood using forward primer 5′ TGGACAAGCTCTTACGTCCT and reverse primer 5′ CCCAGGGATTGGAAATGCAG. Family 4 Sanger sequencing was performed at UCL using forward primer 5′ ACCTAACATCCCAAGCCTGA and reverse primer 3′ GGGTCTCTGATGGGCAAGTT.

Clinical phenotypes were provided by collaborating physicians with additional information extracted from patient photos, videos, and MRI images. Facial features were assessed by C.I.G.-M. Videos from families 1 and 4 were reviewed by pediatric movement disorder neurologist (M.C.K.). Informed consent was obtained by the treating clinician under their local IRB protocol.

### Gene expression analysis from publicly available data

Expression data from normal tissues was obtained from Genotype-Tissue Expression Project for humans and RIKEN FANTOM5 project for mice. Single-cell brain transcriptomics data were obtained from Allen brain cell atlas (*59*, *60*). Analysis was made using modifications on the python scripts provided by the Allen Institute (https://github.com/AllenInstitute/abc_atlas_access).

### Magnetic resonance imaging

For patients, multiplanar, multisequence MRI of the brain without contrast: Sagittal T1 FLAIR, axial T2, sagittal T2 CUBE, sagittal FLAIR CUBE, axial FLAIR, axial T2*, axial DWI, coronal T1 IR 3 plane reconstructions. Patient 1-1 acquired at ages 7 and 10 years, patient 1-2 acquired at age 26 months, patient 5-1 at age 3 years, and patient 5-2 at 21 months. Neuroimaging findings were also assessed and measured by a board-certified neuroradiologist (P.C.) (*61*). Corpus callosum (*61*) and cerebellar measurements (*62*) were compared to age and sex matched controls to determine whether they fell below a threshold of the third percentile.

Several image processing steps were performed on the T1-weighted brain MRIs (*55*) including registration to the Colin 27 Average Brain Atlas, correcting image bias using the N4 algorithm, followed by intensity normalization and image denoising, using anisotropic diffusion. Skull stripping was performed using an in-house algorithm developed in Python. In this approach, intradural cerebrospinal fluid was identified using thresholding and morphological operations, following which the lateral ventricles were isolated on the basis of their spatial location, allowing the volume of the lateral ventricles (in milliliters) to be extracted. Cerebral brain tissues (gray matter, white matter) were then isolated on the basis of their MR intensities using the expectation maximization and Markov random field approach. From the cortical gray matter segmentation, three measures of cortical shape were measured (cortical thickness, curvature, and sulcal depth) to quantify shape abnormalities. Measures were converted to a *z* score (a measurement of SD from normal population) from healthy cortical shape measures measured from the corresponding cortical region compared to the Child Mind Institute Healthy Brain Network cohort of 564 typically developing children (Eq. 1), based on cortical regions from the Automated Anatomical Labeling atlas

$$z-{\mathrm{score}}_{\text{subject}}=\frac{\left(x_{\text{subject}}-\mu_{\text{TDC}}\right)}{\sigma_{\text{TDC}}}$$ (1)

One MRI from each of the two patients in family 1 passed the quality checks for initial MRI data quality and processed segmentations. For each participant, *z* scores of gray matter volume, white matter volume, ventricle asymmetry (Eq. 2), were extracted

$$\text{Ventricle asymmetry}=\frac{\left({\mathrm{vol}}_{\text{left}}-{\mathrm{vol}}_{\text{right}}\right)}{\left({\mathrm{vol}}_{\text{left}}+{\mathrm{vol}}_{\text{right}}\right)}$$ (2)

For mice, high-resolution Mn-enhanced mouse brain three-dimensional (3D) images were acquired on a 9.4-T BioSpec scanner equipped with 114-cm gradient coil (maximum gradient strength 530 mT/m; Bruker Biospin Corp., Billerica, MA). A Bruker ID 4-cm quadrature volume coil was used for radio frequency excitation and detection. Twenty-four hours before imaging, mice were injected intraperitoneally with MnCl_2_ at a dose of 0.5 mmol/kg. During imaging sessions, mice were anesthetized with 1 to 2% isoflurane gas in air and were positioned prone in the scanner. Animal body temperature was maintained with a circulating warm water bath and animal respiration was monitored with an animal physiological monitoring system (SA Instruments Inc., Stony Brook, New York). First, T2-weighted scout brain images along with three orthogonal orientations were acquired. Then, 3D T1-weighted mouse brain images along the transaxial orientation were acquired covering the whole brain using fast low-angle shot gradient echo sequence with the following acquisition parameters: repetition time, 22 ms; echo time, 3.6 ms; and an isotropic spatial resolution of 100 μm and total imaging time of 2 hours.

### Plasmid transfection

HEK293T cells were transfected with pD649–internal ribosomal entry site (IRES)–green fluorescent protein (GFP) (empty vector), pD649-3xFLAG-PGBD5-IRES-GFP encoding WT *PGBD5*, or plasmids encoding *PGBD5* c.49#G>T, p.(Glu17*) mutation from family 1 or *PGBD5* c.509del, p.(Phe170Serfs*5) mutation from family 2. Briefly, 100,000 HEK293T cells were seeded in a six-well plate on day 0. The next day cells were transfected with 1 μg of expression plasmids using TransIT-LT1 (MirusBio MIR 2304) following the manufacturer’s instructions. Sixteen hours posttransfection, the media were replaced with fresh media. Seventy-two hours posttransfection, the cells were trypsinized and pelleted for protein extraction.

### Western immunoblotting

Cells were lysed using Covaris sonication in radioimmunoprecipitation assay lysis buffer. Extracted protein was then quantified using the Pierce BCA assay (Thermo Fisher Scientific, A65453). Briefly, 30 μg of protein extract was separated using NuPAGE 4 to 12% gradient gel (Invitrogen NP0322PK2), and electrophoresis was performed at 120 V. Proteins were then transferred to a polyvinylidene difluoride membrane (Millipore Sigma, IPVH00010) using 20% methanol transfer buffer at 30 V for 1.5 hours at 4°C. The membrane was then blocked with 5% nonfat milk in TBST and then incubated overnight at 4°C with one of the following primary antibodies: FLAG antibody (Millipore Sigma, F1804), GFP antibody (Thermo Fisher Scientific, MA5-15256), and ACTIN antibody (Cell Signaling Technology, 3700). The blots were then incubated with horseradish peroxidase (HRP)–conjugated secondary antibody (Sigma-Aldrich NA931) for 1 hour at room temperature (RT) and imaged after incubation with SuperSignal West Atto (Thermo Fisher Scientific, A38555) using the IQ800 imager (GE).

### Animal handling

All animal procedures were performed following the guidelines of the Institutional Animal Care and Use Committee and approved by the Research Animal Resource Center of the Memorial Sloan Kettering Cancer Center. All mice were housed in groups with up to five animals per cage with a 12-hour light/dark cycle, starting at 06:00 a.m. Food and water were available ad libitum.

### Genetic engineering of *Pgbd5*-deficient mice

To generate *Pgbd5*-deficient mice, we used the dual recombinase-mediated cassette exchange strategy (*26*). First, *tm1e* vector targeting exon 4 of mouse *Pgbd5* (Knockout Mouse Programme) was electroporated in C57BL/6 embryonic stem (ES) cells, followed by their microinjection into Balb/c blastocysts (Ingenious Targeting Laboratory). Resulting *tm1a* chimeras with a high percentage black coat color were mated to C57BL/6 FLP mice expressing the Flp recombinase to remove the Neo cassette to generate *tm1c Pgbd5*-floxed mice, as confirmed using genotyping with SC2 and SC4 primers (SC2: GAGAGCACCGTTGGTGCATATCAG, SC4: AGAGTATGAGCGGGAGAGGAGCAG). *Pgbd5*-floxed mice were crossed to B6.FVB-Tg(EIIa-cre)C5379Lmgd/J (EIIa-Cre) mice to generate *tm1d Pgbd5*–deficient mice, as confirmed by genotyping with SC2 and SC4 primers. *Pgbd5*-deficient mice were backcrossed to C57BL/6J mice for six generations. *Pgbd5^fl/fl^* mice are available from the Jackson Laboratory (strain 037535).

### Genetic engineering of *Pgbd5* catalytic–deficient mice

To generate *Pgbd5* aspartate mutant mice, we used CRISPR-Cas9–assisted genome editing by zygote microinjection in C57BL/6J (the Jackson Laboratory, ME, USA). Briefly, mice of 3 to 6 weeks were used as zygote donors. Fertilized eggs were recovered at pronuclear staged from oviducts of copulated females. Zygotes were microinjected in a drop of KSOM medium. Injection cocktails consisted of Cas9 protein (100 ng/μl; PNABio), in vitro–synthesized Cas9 mRNA (50 ng/μl; kit, company), CRISPR RNA (crRNA)(s) (50 ng/μl each; Integrated DNA Technologies), trans-activating CRISPR RNA (tracrRNA) (200 ng/μl; Integrated DNA Technologies), and donor single-stranded DNA(s) (20 ng/μl each; Integrated DNA Technologies). Cocktails were mixed just before microinjection and kept on ice. Guide RNA sequences were designed by CRISPR tools (IDT, https://idtdna.com/site/order/designtool/index/CRISPR_SEQUENCE and ChopChop https://chopchop.cbu.uib.no/). The crRNA and tracrRNA sequences were as follows; crRNA 1 for D/A on exon 2, sequence (AGCCACTCTGCAGGGAGTCG), crRNA 2 for D/A on exon 3, sequence (ACATGAACCCCTGATTGACG); tracrRNA. The microinjection setup was composed of an inverted microscope (TE200-U, Nikon), microinjectors (CellTram vario, Eppendorf; Femtojet, Eppendorf) and manipulators (TransferMan 4r, Eppendorf). Injection cocktails were injected into pronuclei, and after injection, zygotes or overnight-cultured two cell–stage embryos were surgically implanted into the oviduct of B6/CBA F1 females primed for pseudopregnancy by mating with vasectomized males. Offspring born from the implanted embryos (the founders) were screened for the presence of the insertion of the donor sequences by PCR of genomic DNA extracted from toe clips. PCR primers used are as follows; primer #1 D/A exon 2 (AGGCTTCTATAGCAACCGGAGCC); primer #2 exon 2 (TGCATGCATGGACCTGCGTGTGG); primer #3 exon 3(AGACTCCTGGTCAGAGAAGTCAG); primer #4 D/A exon 3 (TGATGAACCCGGTTGAAGAGC). *Pgbd5^D236A^* mutant mice are available from the Jackson Laboratory (strain 038881).

### Genetic engineering of *Pgbd5^3xFlag-HA-P2A-eGFP^* knock-in mice

*Pgbd5^3xFlag-HA-P2A-eGFP^* mice were generated by targeting iTL BF1 (C57BL/6 FLP) ES cells (inGenious Targeting Laboratory). A targeting vector was designed to comprise a *3xFlag-HA-P2A-eGFP* in the upstream of the stop codon in exon 7 (fig. S4A). Targeted ES cells were microinjected into the Balb/c blastocysts. Resulting chimeras were mated to C57BL/6N mice to generate germline transgenic mice. The resulting mice were backcrossed to C57BL/6J mice to eliminate the *FLP* allele. Probe sets were designed to detect *7WT* (72 bp) and *7MD* (254 bp) (fig. S4A) for genotyping animals (Transnetyx). *Pgbd5^3xFlag-HA-P2A-eGFP^* knock-in mice are available from the Jackson Laboratory (strain 039713).

### Immunofluorescence analysis of *Pgbd5^3xFlag-HA-P2A-eGFP^* expression

Under deep anesthesia, 3-month-old *Pgbd5^3xFlag-HA-P2A-eGFP^* homozygous and C57BL/6J WT mice were perfused with intracardiac 0.9% saline followed by 4% paraformaldehyde (PFA)/0.1 M phosphate buffer (PB). Brains were dissected and further fixed in 4% PFA/0.1 M PB overnight, cryoprotected in 30% sucrose, and embedded in optimal cutting temperature (OCT) compound. Blocks were sectioned sagittally in 10-μm sections using a cryostat (Leica). Sections were stored at −20°C. Enhanced green fluorescent protein (eGFP) signal together with 4′,6-diamidino-2-phenylindole (DAPI) was imaged using LSM800 confocal microscope (Zeiss). Thereafter, antigen retrieval was done in sodium citrate buffer [10 mM sodium citrate and 0.05% Tween20 (pH6.0)] for 1 hour at 99°C. Anti-NeuN rabbit monoclonal antibody (D4G40: Cell Signaling Technology) was used in combination with an Alexa Fluor 647 F(ab′)_2_ fragment donkey anti-rabbit IgG (H + L) (Jackson Immuno Research). Two confocal images for eGFP and NeuN were coregistered using Photoshop (Adobe). For staining GFAP and TMEM119, anti-GFAP (GA5; Millipore Sigma) and anti-TMEM119 antibody (28-3; Abcam) were used without antigen retrieval, respectively. Images were captured simultaneously for eGFP and GFAP, or eGFP and TMEM119.

### Mouse genotyping

DNA was extracted from tail clips using PureLink Genomic DNA kit (K182000, Invitrogen) following the manufacturer’s instructions. PCR was performed using SC2 and SC4 primers and Platinum PCR SuperMix High Fidelity (12532016, Invitrogen) reagents, following a protocol of 30 cycles that consists of denaturation at 98°C for 10 s, annealing at 55°C for 30 s, and elongation at 72°C for 30 s. After the 30 cycles, an additional elongation step at 72°C for 10 min was performed.

### Brain weight assessment

Mice were euthanized following IACUC specifications at 60 days old. Afterward, brain (from olfactory bulbs to medulla oblongata) was extracted and weighted on a precision balance scale.

### Behavioral studies

All tests were conducted using 8- to 12-week-old mice. During all behavioral tests, the investigators who performed the tests were blinded to experimental genotypes. Behavioral tests were conducted during the light phase, between 10:00 hours and 16:00 hours. On test days, the animals were transported to the dimly illuminated behavioral laboratory and left undisturbed for 1 hour before testing.

### Locomotor assay

The locomotor assay used a 15 inch–by–21 inch (38.1 cm–by–53.34 cm) black box, divided into 12 even-sized [(4 × 3 inch) 10.16 cm–by–7.64 cm] rectangles. The time spent and distance traveled in the two rectangles at 150 lux of light illumination were recorded and analyzed using Med Associates Inc. automated tracking system, according to the manufacturer’s instructions (Med Associates Inc). At least five mice per group were used in this assay.

### Elevated plus maze

The EPM used a cross maze with 12 inch–by–2 inch (30.48 cm–by 5.08 cm) arms with 50 lux of light illumination. Animals were introduced to the middle portion of the maze facing an open arm and allowed to freely explore for 10 min. Time spent and distance traveled in the open and closed arms were measured by an automated video-tracking system (Noldus Information Technology). Data were analyzed for distance traveled in the open arm because it is least confounded by repeated entry. At least five mice per group were used in this assay.

### Rotarod test

An accelerating rotarod was used with 3-cm cylinders (47650, Ugo Basile). Each experiment included a training phase that included four trials with 15 min between trials. During training, the animals were placed on the rod and allowed to run until the speed reached 5 rpm, and then the rod was accelerated from 5 to 40 rpm over the course of 300 s, followed by two trials for 60 s at 4 rpm, with 10 min between all trials. Mice able to remain on the rod for the training phase were assayed, following 30 min of rest in a cage. The final assay did not include the training phase. Only alternate rod positions were used to minimize the confounding effect of neighboring animals. At least nine mice per group were used in this assay.

### Grip strength assay

Mice were assessed for grip strength performance immediately after the rotarod test. Grip strength was measured as tension force using the force gauge (1027SM Grip Strength Meter with Single Sensor, Columbus Instruments). To assess forelimb grip strength measurement, the mice were held by the base of their tails over the top of the grid so that only front paws were able to grip the grid platform T-bar. With the torso in a horizontal position, the mice were pulled back steadily until the grip was released while measuring the grip force. Grip strength measurements for both sets of limbs were performed similarly, with the torso held parallel to the grid for forelimb and hindlimb measurements. Measurements were performed five times with 5-min resting periods. At least nine mice per group were used in this assay.

### Seizure assessment

For seizure analysis, we used the Racine scale, as described previously (*63*). Briefly, the mice were manually suspended by their tails while rotated 10 times for 5 s, after which they were placed individually into empty cages for observation. Each mouse was observed for 2 min.

### Waterbath tail immersion

Using a water bath heated at 47°C, 1 cm of the tail tip was submerged, and time to tail flick was measured. Five mice per group were used in this assay.

### Tissue perfusion and fixation

Mice were anesthetized using ketamine and xylazine, followed by midline sternotomy. Upon cannulation of the left heart ventricle and connection to the Masterflex C/L peristaltic pump (Antylia Scientific), mice were perfused for 2 min with phosphate-buffered saline (PBS), followed by 6 min with 4% (w/v) PFA in PBS. Dissected tissues were fixed in 4% methanol-stabilized PFA (15714-S, Electron Microscopy Sciences) in PBS for 16 hours at 4°C. Upon anesthesia of pregnant dams and externalization of the uterus, the embryos were dissected and immobilized on 6-cm plates using pin needles (Silicone Dissecting Pad Kit 501986, World Precision Instruments). Embryos were perfused with 0.5 ml of PBS, followed by 1 ml of 4% methanol-stabilized PFA (15714-S, Electron Microscopy Sciences) in PBS. Fixed tissues were washed in deionized water twice and stored in 70% aqueous ethanol followed by paraffin embedding or incubated in a 30% (w/v) sucrose in PBS and frozen in Clear O.C.T. compound (4585, Thermo Fisher Scientific).

### Immunofluorescence and histology

Fixed specimens (at least three per group) were sectioned using a microtome (Leica RM2155) in 5-μm sections. Slides were deparaffinized and rehydrated using equal volumes of xylene (100%) three times followed by a graded alcohol series (100 to 50%), followed by washing with deionized water. Antigen retrieval was performed using two methods: for PGBD5 antibody, proteinase K at 10 U/ml, or for the other antibodies, citric acid–based solution (H-3300, Vector) in a 95°C water bath for 30 min, followed by 5-min immersion in RT solution. Sections were then washed twice with PBS (0.1 M, pH 7.5) and once with PBST (PBS with 0.25% Triton X-100). Slices were subsequently blocked for 30 min at RT using blocking solution: 10% normal donkey serum (NSD, 017-000-121, Jackson ImmunoResearch Laboratories) and 1% bovine serum albumin (BSA, A2153, Sigma-Aldrich) in PBST. Primary antibodies (table S7) were incubated for 16 hours at 4°C in blocking solution. Slides were washed four times for 10 min using PBST at RT. Secondary antibodies (table S7) were incubated for an hour in the dark at RT in 1% NSD and 1% BSA in PBST. Slides were washed four times in the dark using PBST for 10 min. Slides were then stained with Hoescht-33342 (H1399, Invitrogen) for 5 min. Slides were washed one last time for 5 min in PBS, and coverslips were mounted at medium light using ProLong Diamond antifade mountant containing DAPI (P36962, Thermo Fisher Scientific). Sagittal sections of cerebellum were stained with hematoxylin and eosin.

### TUNEL assay

The same deparaffinization and rehydration protocol from immunofluorescence was used. Click-iT TUNEL assay was performed following the manufacturer’s instructions (C10617, Invitrogen).

### RNA in situ hybridization

Paraffin-embedded tissue sections were cut at 5-μm thickness and kept at 4°C. Samples were loaded into Leica Bond RX, baked for 30 min at 60°C, dewaxed with Bond Dewax Solution (Leica, AR9222), and pretreated with EDTA-based epitope retrieval ER2 solution (Leica, AR9640) for 15 min at 95°C (no proteolytic retrieval). For fluorescence in situ hybridization (FISH), the probe for mouse *Pgbd5* [Advanced Cell Diagnostics (ACD), catalog no. 561948] was hybridized for 2 hours at 42°C. Mouse *PPIB* (ACD, catalog no. 313918) and *dapB* (ACD, catalog no. 312038) probes were used as positive and negative controls, respectively. The hybridized probes were detected using RNAscope 2.5 LS Reagent Kit – Brown (ACD, catalog no. 322100) according to the manufacturer’s instructions with some modifications (DAB application was omitted and replaced with fluorescent Alexa Fluor 488/tyramide (Invitrogen, B40953bak) for 20 min at RT). FISH slides were loaded in Leica Bond RX for immunofluorescence staining. Samples were pretreated with EDTA-based epitope retrieval ER2 solution (Leica, AR9640) for 20 min at 95°C. Mouse monoclonal antibody Tuj1 (BioLegend, MIMS-435P) was incubated for 1 hour at RT. Samples were then incubated with Leica Bond Post-Primary reagent (Rabbit anti-mouse linker) [included in Polymer Refine Detection Kit (Leica, DS9800)] for 8 min, followed by incubation with Leica Bond Polymer (anti-rabbit HRP) [included in Polymer Refine Detection Kit (Leica, DS9800)] for another 8 min. Alexa Fluor tyramide signal amplification reagents (CF 594/ tyramide conjugates (Biotium, 92174) were used for signal visualization. After staining, slides were washed in PBS and incubated in DAPI (5 μg/ml; Sigma-Aldrich) in PBS (Sigma-Aldrich) for 5 min, rinsed in PBS, and mounted in Mowiol 4–88 (Calbiochem). Slides were kept overnight at −20°C before imaging.

For RNA in situ hybridization, BaseScope hybridization probes specific for Pgbd5 exon 4 were generated as per the manufacturer’s instructions (ACD, catalog no. 1181898-C1). Upon cardiac perfusion and fixation in 4% PFA/0.1 M PB overnight, dissected brains were washed twice with 30% sucrose/PBS and incubated overnight at 4°C. After cryopreservation in sucrose, the brains were embedded in OCT (Thermo Fisher Scientific), and blocks were sectioned sagittally in 10-μm sections using a rotary microtome cryostat (Leica). Sections were stored at −80°C. Cryosections were baked for 1 hour at 60°C and fixed in 4% PFA for 15 min followed by washing in PBS. After dehydration, epitope retrieval treatment with ER2 for 5 min at 95°C and subsequent protease III treatment for 15 min at 40°C were performed. The probe set was hybridized for 2 hours at 42°C. Signal amplification steps were performed according to the manufacturer’s protocol. Fast Red (Leica Bond Polymer Refine Red Detection kit, DS9390) was used as the chromogen. Hematoxylin was used as a counterstain. Mouse Ppib (ACD, catalog no. 701078) and *Bacillus subtilis* dapB (ACD, catalog no. 701018) probe sets were used as positive and negative controls, respectively.

### Flow cytometry analysis

Thymus and spleen were collected using standard dissection. Lymphocytes were extracted by mechanical dissociation followed by erythrocyte lysis using red blood cell lysis buffer (BioLegend, catalog no. 420301). Cells were filtered using a 70-μm mesh (Fisherbrand, catalog no. 22-363-528). Isolated cells were blocked on ice for 30 min using mouse IgG isotype control antibodies (Invitrogen, catalog no. 10400C) at 1:1000 dilution in 2.5% fetal bovine serum in PBS. Cells were aliquoted at 1 × 10^6^ cells/ml. Antibodies were added at the specified concentrations (table S7) and incubated for 30 min on ice protected from light. Nuclear staining was done using DAPI. Flow cytometry analysis was performed using BD LSRFortessa cell analyzer and FCS Express 6 software.

### Microscopy and image acquisition

Confocal imaging was performed using a Leica TCS SP5 microscope with plan-apochromat 20×/0.75 numerical aperture (NA) objective lens with 4× digital zoom. Images were quantified using blinded observers. Epifluorescence and bright-field images were acquired using 20×/0.8 NA objective from the Pannoramic P250 Flash microscope (3Dhistech).

### Whole-genome sequencing

Matched littermates (three per group) were dissected, and then, tissue was extracted, flash frozen, and kept at −80°C till further processing. For adults, olfactory bulb, hippocampus, cerebellum, and PBMCs were obtained. For embryos, forebrain, midbrain, hindbrain, and liver were collected. After PicoGreen quantification and quality control (QC) by Agilent BioAnalyzer, 137 to 500 ng of genomic DNA was sheared using a LE220-plus focused ultrasonicator (Covaris, catalog no. 500569) and sequencing libraries were prepared using the KAPA Hyper Prep Kit (Kapa Biosystems KK8504) kit with the following modifications to the manufacturer’s instructions. After adapter ligation, the libraries were subjected to 0.5× size selection using AMPure XP beads (Beckman Coulter, catalog no. A63882). DNA quantity was estimated by real-time PCR using sequencing adapter primers, and libraries were mixed at equimolar ratios for sequencing. Samples were sequenced using NovaSeq 6000 in a 150 bp/150 bp paired-end mode, with the NovaSeq 6000 SBS v1 kit and S4 flow cells (Illumina).

### Sequenced data alignment and processing

All FASTQ files corresponding to the same sample were merged. Sequencing reads were aligned to the mouse reference genome (GRCm38/mm10) downloaded from https://genome.ucsc.edu using bwa-mem algorithm (*64*). We used bammarkduplicates to mark the duplicated reads. For the alignment summary metrics, we used Alfred v0.1.16 (*65*).

### Variant calling

Variant calling was performed using Pindel version 2.2.3 (*39*, *66*) and Delly2 (*37*) with the following parameters: -c 0.05, -a 0.05, and -m 15. Following the merging of results, duplicate calls were filtered using a similarity window of 300 bp, keeping those with the highest detection quality, variant allele frequency. Only variants with features exceeding default PASS quality filter were included for analysis.

### Identification and analysis of somatically rearranged genes

For the detection of genes affected by observed variants, we used the BEDTOOLS package (*67*) and the annotation of National Center for Biotechnology Information genes for mouse downloaded from https://genome.ucsc.edu. To study the effect of the different subsets of variants, we used the ENSEMBL Variant Effect Predictor (https://ensembl.org/Tools/VEP) (*68*) with default settings.

### Identification and analysis of somatic genome rearrangement regions

For the detection of genomic intervals, the regions have been created dynamically through the list of mutations contained in each group, with a static window size of 3 Mb, a minimum of two mutations per window, and excluding groups of mutations that come from larger windows using Intersect in bedtools (*67*).

### Recurrence analysis of polyclonal somatic DNA rearrangements

A series of R-scripts were written to first parse and filter the VCF files for events, which are available from https://github.com/kentsisresearchgroup/P5BrainReorg. The filtered events were then checked for overlaps, and clusters of mutually overlapping groups were output. For the events called by the Delly2 algorithm, the overlaps were computed in two ways. Method 1 looked at the actual genomic intervals, and two events were defined to overlap if either the length of both was less than 100,000 bp or the percent overlap was greater than 0.1%, where the percent overlap was computed as the length of the overlap divided by the average size of the two events. Method 2 examined the overlap between the break points of the somatic variants. The ends were defined as a region of 701 bp centered on each end of the event. It was these edge regions that were then intersected for overlaps, this time using the rule that the percent overlap was greater than 1%.

Once all overlapping events were identified, clusters were determined by looking for groups of events that all overlapped with each other. That is each element in the cluster had to overlap with every other element, as defined as a maximal clique in graph theory. We used the R package igraph to both construct the overlap graph and find all maximal cliques in the graph. We then filtered for sets with three or more events and output the table of clusters. To examine how the clusters varied on the filtering parameters this entire procedure was repeated multiple times each time adjusting one of the following parameters: normalized total read counts (RC/EventSize), high-quality variant junction reads (RV), variant allele frequency [RV/(RR + RV), where RR is the high-quality reference junction reads].

For SNV and small insertions/deletions called by Mutect2, a similar procedure was used to identify overlaps for the precise same event in multiple samples. Filtering was done on the following variables: allele depth, variant allele frequency, and total depth. Since Mutect2 does not report total depth per sample, we computed an estimate of total depth by taking the maximum value of the following three estimates: RD/(1-AF), AD/AF and AD + RD, where RD is the depth of the reference reads, AD is the depth of the alternate reads and AF was Mutect2’s reported allele frequency. We also computed a consistent variant allele frequency of AD/(AD + RD).

To manually inspect that these rearrangements we extracted split reads and discordant pair-end reads using SAMBLASTER (*69*). We then used SparK with default -pr -cf -o -gl, and mm10 reference gene gtf file for -gtf and -gs “yes” parameters to visualize and plot the split reads found in the regions of interest (*70*).

### Single-nucleus RNA and ATAC sequencing

Brains from 21 days old *Pgbd5^wt/wt^* (*n* = 3) and *Pgbd5^−/−^* (*n* = 3) littermates were extracted. Motor cortices were dissected, flash frozen, and stored at −80°C. Nucleus extraction was performed according to the protocol from Masilionis *et al.* (*71*) using the Singlulator100 (S2 Genomics). Extracted nuclei were stained with 7-aminoactinomycin D (Invitrogen) and FAC-sorted for further nuclei purification. Ten thousand nuclei were targeted for library construction using ChromiumNextGEM Multiome ATAC + Gene Expression kit (10x Genomics).

Cell Ranger ARC v2.0.0 (10x Genomics) was used (“count” option with default parameters) to filter and align raw reads, identify transposase cut sites, detect accessible chromatin peaks, call cells, and generate raw count matrices for scMultiome samples. Alignment was performed using the mm10 reference genome build coupled with the Ensembl 98 gene annotation. Reads that mapped to the intronic regions were excluded for the RNA modality.

QC and subsequent data processing steps were performed using Signac v1.3.0 (*72*) and Seurat v4.3.0 (*73*). QC metrics for RNA and ATAC modalities were calculated independently but were jointly used to filter cells. A combination of thresholds was established for each sample based on hard cutoffs or on the distribution of each metric within the sample (thresholds specified in table S5). In the RNA modality, cells were filtered on the number of genes, unique molecular identifiers (UMIs), and mitochondrial content. In the ATAC modality, cells were filtered on the number of peaks detected, transcription start site enrichment, and nucleosome signal.

### Cell type annotation in single-nucleus RNA data

Annotation of cell types was performed using a consensus approach between seven different reference-based annotation tools, using two references separately. Six machine learning–based prediction tools [ACTINN (*73*), scAnnotate (*74*), SciBet (*75*), SingleR (*76*), scClassify (*77*), and Support Vector Machines] and one model based on maximum Spearman correlation [as previously described (*43*) were trained on a developmental murine atlas of the forebrain (*43*) or a postnatal murine atlas of the isocortex with high neuronal diversity (*42*)]. Labels predicted by each tool were aggregated into major cell type classes according to the ontology found in table S6, and a consensus label annotation was assigned to each cell using a majority vote approach between tools (with at least two tools agreeing). Predictions based on the developmental atlas (*43*) were used to assign broad cell types, while predictions based on (*42*) were used to annotate neuronal types.

### Normalization, dimensionality reduction, and doublet detection in single-nucleus multiome data

For the RNA modality, libraries were scaled to 10,000 UMIs per cell and log normalized. UMI counts and mitochondrial content were regressed out from normalized gene counts and the residual *z*-scored gene-wise. Dimensionality reduction was performed using principal components analysis on the top 2000 most variable features. For the ATAC modality, peaks were called using MACS2 (v2.2.7.1) (*78*) using the CallPeaks function from Signac library with default parameters. ATAC reads were quantified in each peak per cell, and a resulting count matrix was generated. Dimensionality reduction was performed using latent semantic indexing (LSI) (*79*).

A weighted nearest neighbor graph was constructed between all cells using the first 30 principal components of the RNA data and the top six dimensions of the LSI reduction from the ATAC data with the following default parameters: 20 multimodal neighbors, 200 approximate neighbors, and L2 normalization enabled. This weighted nearest neighbor graph was used as input for clustering using a shared nearest-neighbor (SNN) algorithm (*73*) based on the Louvain algorithm on a *k*-nearest neighbor graph with *k* = 20 and resolution 0.2. For each sample, doublets were identified using scDblFinder v1.8.0 (*80*) with the recommended cluster-based approach and with the RNA modality counts.

### Joint sample visualization in single-nucleus RNA data

For visualizing multiple samples, samples were first merged by genotype, generating a joint sample object for knockout (KO) and WT separately, as follows. Corresponding RNA libraries were merged without batch correction, followed by scaling, normalization, and dimensionality reduction as described in the previous section except for the regression of variables. The first 30 principal components were used as input for projection into two dimensions [Uniform Manifold Approximation and Projection (*81*)] and for clustering (SNN) with *k* = 20 and resolution 0.5. These genotype joint objects were used to perform postclustering QC (described in the next section). After postclustering QC, all samples were merged in a single joint object (in the same manner as the merging of genotype joint objects) to perform comparison of cell populations, differential gene expression analysis, and differential promoter activity analysis between KO and WT samples.

### Postclustering QC

QC of cell populations and clusters in each genotype joint object was performed to avoid an imbalance in cell populations between KO and WT samples. First, clusters consisting of doublets with a proportion greater than 70% were completely removed. Cells called doublets were filtered in the remaining clusters.

Next, cell type annotations were inspected at the cluster level as follows. Cluster annotations consisting of a mixture of conflicting cell lineage labels were manually inspected by assessing their gene signatures. Gene signatures were derived by identifying DEGs in each cluster compared to all other cells using Seurat’s FindAllMarkers function (Wilcoxon rank sum test). For each cluster, positive markers were sorted by adjusted *P* value, mitochondrial (defined as having gene symbols starting with “mt-”) and ribosomal genes (defined as having gene symbols matching “Rps,” “Rpl,” “Mrps,” and “Mrpl”) were filtered out, and the top 100 genes were used as a signature. Some clusters were then reannotated as “striatal neurons” or “other neurons” by assessing the expression of the cluster gene signatures in a whole adult mouse brain atlas (*59*).

Last, clusters driven by a single sample (nonreproducibility across replicates likely due to sampling issues), were removed only if a similar cell type population was not observed in the opposing genotype integration. Clusters that were manually annotated and retained after postclustering QC can be observed in tables S8 (WT) and S9 (KO).

### Comparison of cell population proportions

To identify significant differences in cell population proportions between different genotypes, a permutation test was applied using the scProportionTest package v0.0.0.9 (*82*) with a total of 10,000 permutations. Only cells of known cortical origin (excluding striatal neurons, other neurons, and “no consensus” cells) were considered for comparison of cell population proportions.

### Differential expression in single-nucleus RNA data

Differential expression analysis was performed in a per-cluster basis within the joint sample object. Only clusters with greater than 200 cells were considered. First, in each cluster, if the proportion of KO or WT cells was below 40%, random downsampling of cells was performed to balance the number of cells per genotype. Next, a Wilcoxon rank sum test was applied through the FindMarkers function from the Seurat package (logFC.threshold = 0, min.pct = 0.05). Gene ontology pathway analysis was performed on each cluster’s DEGs with adjusted *P* values <0.05 using the enrichGO function from the clusterProfiler package v4.0.0 (*83*), using all detected genes in the integration as the universe (OrgDb = org.Mm.eg.eb, keyType = “SYMBOL,” ont = “BP”).

### Differential promoter activity in single-nucleus ATAC data

Quantification of chromatin accessibility was performed on a per-gene basis by counting fragments that overlapped the gene promoter region, defined as 5-kb bins centered on the transcription start site. Next, differential promoter activity analysis was performed in a per-cluster basis. Using the same clusters as in the differential expression analysis, random downsampling of cells per genotype was performed if necessary due to imbalanced cell numbers. Last, differential promoter activity between genotypes was calculated using a Wilcoxon rank sum test through the FindMarkers function from the Seurat package (logFC.threshold = 0, min.pct = 0.05).

### Statistical analysis

Statistical analyses were performed using OriginPro and R. Statistical significance was estimated using two-tailed *t* tests for continuous pair variables, two-tailed Fisher exact and χ2 tests for discrete variables, and one and two-way ANOVAs with Tukey’s test and Bonferroni or Dunn-Šidák correction for multiple comparisons. In addition, we analyzed immunofluorescence images using a generalized linear mixed model with a binomial distribution to account for variability across multiple regions within individual mice and differences in cell counts per region. The models were fitted using the Laplace approximation method implemented by the glmer function in R, and the significance of differences was determined by Wald tests (*84*).

## Supplementary Materials

### The PDF file includes:

Legends for tables S1, S3 to S6, S8 and S9 Tables S2 and S7 Figs. S1 to S32 Data S1, S3 and S4 Legend for data S2
